# Supplementary material for: Effect of different lithological assemblages on shale reservoir properties in the Permian Longtan Formation, southeastern Sichuan Basin: Case study of Well X1
Source: PLoS One. 2022 Aug 12;17(8):e0271024. doi: 10.1371/journal.pone.0271024 (PMC9374248; doi:10.1371/journal.pone.0271024)
Supplement: Attachment — Submitted filename: PONE-D-21-40287_Anas Abbassi_Reviewer_Attachment.pdf [file pone.0271024.s007.pdf]

PLOS ONE

Effect of different lithological assemblages on oil shale reservoir properties in the Permian Longtan Formation, southeastern Sichuan

--Manuscript Draft--

**Manuscript Number:**	PONE-D-21-40287
**Article Type:**	Research Article
**Full Title:**	Effect of different lithological assemblages on oil shale reservoir properties in the Permian Longtan Formation, southeastern Sichuan
**Short Title:**	Effect of different lithological assemblages on oil shale reservoir properties
**Corresponding Author:**	Qian CAO<br>State Key Laboratory of Shale oil and Gas Enrichment Mechanisms and Effective Development<br>Chengdu, Sichuan CHINA
**Keywords:**	Permian Longtan Formation; southeastern Sichuan Basin; lithological assemblages; shale reservoir characteristics
**Abstract:**	Various types of marine–continental transitional facies are present in the gas-bearing shales of the southeastern Sichuan Basin. A review of the different lithological assemblages in these rocks is important for assessing the likely shale gas content and the development of the storage space. This study of the lithological assemblages of the Permian Longtan Formation in the southeastern Sichuan Basin at Well X1 used core observations, optical thin-section observations, Ar-ion polishing, scanning electron microscopy and nitrogen adsorption tests to compare and analyze storage space types and pore structures in the shale to determine the sedimentary paleoenvironment, petromineralogy and organic content properties. The marine–continental transitional facies in the study area were deposited in a warm climate that favored enrichment by organic matter. The kerogen is type I (average vitrinite reflectance 2.66%), which is within the favorable thermal maturity range for the presence of shale gas. The lithology mainly comprises shale, siltstone and limestone (with bioclasts), and a coal seam. The lithological development divides the Longtan Formation into lower (swamp), middle (tidal flat/lagoon) and upper (delta) sub-members. From lower to upper divisions, the lithofacies evolved from silty shale to clay shale and then to shale intercalated with siltstone or calcareous layers. The proportion of intergranular and dissolution pores in the clay minerals decreases gradually from lower to upper sub-members, and pore size sizes also tend to decrease. Relatively large diameter pores and microfractures occur in the inorganic matter in the lowest sub-member. Quartz and clay are the main constituents of the shale, respectively contributing to the specific surface area and specific pore volume of the reservoir space.
**Order of Authors:**	Qian CAO
	Xin Ye
	Yan LIU
	Ke Jiang
**Additional Information:**	
**Question**	**Response**
**Financial Disclosure**<br><br>Enter a financial disclosure statement that describes the sources of funding for the work included in this submission. Review the submission guidelines for detailed requirements. View published research articles from *PLOS ONE* for specific	The author(s) received no specific funding for this work.

Powered by Editorial Manager® and ProduXion Manager® from Aries Systems Corporation

examples.<br><br>This statement is required for submission and **will appear in the published article** if the submission is accepted. Please make sure it is accurate.<br><br>**Unfunded studies**<br>Enter: *The author(s) received no specific funding for this work.*<br><br>**Funded studies**<br>Enter a statement with the following details:<br>• Initials of the authors who received each award<br>• Grant numbers awarded to each author<br>• The full name of each funder<br>• URL of each funder website<br>• Did the sponsors or funders play any role in the study design, data collection and analysis, decision to publish, or preparation of the manuscript?<br>• **NO** - Include this sentence at the end of your statement: *The funders had no role in study design, data collection and analysis, decision to publish, or preparation of the manuscript.*<br>• **YES** - Specify the role(s) played.<br><br>* typeset	
**Competing Interests**<br><br>Use the instructions below to enter a competing interest statement for this submission. On behalf of all authors, disclose any competing interests that could be perceived to bias this work—acknowledging all financial support and any other relevant financial or non-financial competing interests.<br><br>This statement is required for submission and **will appear in the published article** if the submission is accepted. Please make sure it is accurate and that any funding sources listed in your Funding Information later in the submission form are also declared in your Financial Disclosure statement.	The authors have declared that no competing interests exist.

Powered by Editorial Manager® and ProduXion Manager® from Aries Systems Corporation

View published research articles from *PLOS ONE* for specific examples.<br><br>**NO authors have competing interests**<br><br>Enter: *The authors have declared that no competing interests exist.*<br><br>**Authors with competing interests**<br><br>Enter competing interest details beginning with this statement:<br><br>*I have read the journal's policy and the authors of this manuscript have the following competing interests: [insert competing interests here]*<br><br>* typeset	
**Ethics Statement**<br><br>Enter an ethics statement for this submission. This statement is required if the study involved:<br><br>• Human participants<br>• Human specimens or tissue<br>• Vertebrate animals or cephalopods<br>• Vertebrate embryos or tissues<br>• Field research<br><br>Write "N/A" if the submission does not require an ethics statement.<br><br>General guidance is provided below. Consult the submission guidelines for detailed instructions. **Make sure that all information entered here is included in the Methods section of the manuscript.**	N/A

Powered by Editorial Manager® and ProduXion Manager® from Aries Systems Corporation

**Format for specific study types**<br><br>**Human Subject Research (involving human participants and/or tissue)**<br>• Give the name of the institutional review board or ethics committee that approved the study<br>• Include the approval number and/or a statement indicating approval of this research<br>• Indicate the form of consent obtained (written/oral) or the reason that consent was not obtained (e.g. the data were analyzed anonymously)<br><br>**Animal Research (involving vertebrate animals, embryos or tissues)**<br>• Provide the name of the Institutional Animal Care and Use Committee (IACUC) or other relevant ethics board that reviewed the study protocol, and indicate whether they approved this research or granted a formal waiver of ethical approval<br>• Include an approval number if one was obtained<br>• If the study involved *non-human primates*, add *additional details* about animal welfare and steps taken to ameliorate suffering<br>• If anesthesia, euthanasia, or any kind of animal sacrifice is part of the study, include briefly which substances and/or methods were applied<br><br>**Field Research**<br><br>Include the following details if this study involves the collection of plant, animal, or other materials from a natural setting:<br>• Field permit number<br>• Name of the institution or relevant body that granted permission	
**Data Availability**<br><br>Authors are required to make all data underlying the findings described fully available, without restriction, and from the time of publication. PLOS allows rare exceptions to address legal and ethical concerns. See the PLOS Data Policy and FAQ for detailed information.	Yes - all data are fully available without restriction

Powered by Editorial Manager® and ProduXion Manager® from Aries Systems Corporation

A Data Availability Statement describing where the data can be found is required at submission. Your answers to this question constitute the Data Availability Statement and **will be published in the article**, if accepted.<br><br>**Important:** Stating 'data available on request from the author' is not sufficient. If your data are only available upon request, select 'No' for the first question and explain your exceptional situation in the text box.<br><br>Do the authors confirm that all data underlying the findings described in their manuscript are fully available without restriction?	
**Describe where the data may be found in full sentences. If you are copying our sample text, replace any instances of XXX with the appropriate details.**<br><br>• If the data are **held or will be held in a public repository**, include URLs, accession numbers or DOIs. If this information will only be available after acceptance, indicate this by ticking the box below. For example: *All XXX files are available from the XXX database (accession number(s) XXX, XXX.).*<br>• If the data are all contained **within the manuscript and/or Supporting Information files**, enter the following: *All relevant data are within the manuscript and its Supporting Information files.*<br>• If neither of these applies but you are able to provide **details of access elsewhere**, with or without limitations, please do so. For example:<br><br>*Data cannot be shared publicly because of [XXX]. Data are available from the XXX Institutional Data Access / Ethics Committee (contact via XXX) for researchers who meet the criteria for access to confidential data.*<br><br>*The data underlying the results presented in the study are available from (include the name of the third party*	All relevant data are within the manuscript and its Supporting Information files.

and contact information or URL).<br>• This text is appropriate if the data are owned by a third party and authors do not have permission to share the data.<br><br>* typeset	
Additional data availability information:	Tick here if the URLs/accession numbers/DOIs will be available only after acceptance of the manuscript for publication so that we can ensure their inclusion before publication.

Powered by Editorial Manager® and ProduXion Manager® from Aries Systems Corporation

Manuscript
Click here to access/download;Manuscript;Mnuscript 2021.12.pdf

Effect of different lithological assemblages on oil shale reservoir properties in the Permian Longtan Formation, southeastern Sichuan Basin: Case study of Well X1

Qian Cao[1,2], Xin Ye[1,3], Yan Liu[4], Pengwei Wang[1,3], Ke Jiang[4]

1. State Key Laboratory of Shale Oil and Gas Enrichment Mechanisms and Effective Development, Beijing 100083, China.
2. Shale Gas Evaluation and Exploitation Key Laboratory of Sichuan Province, Chengdu 610091, China.
3. Research Institute of Petroleum Exploration & Production, SINOPEC, Beijing 100083, China.
4. College of Energy and Resources Chengdu University of Technology, Chengdu 610059, Sichuan Province, China.

Abstract: Various types of marine–continental transitional facies are present in the gas-bearing shales of the southeastern Sichuan Basin. A review of the different lithological assemblages in these rocks is important for assessing the likely shale gas content and the development of the storage space. This study of the lithological assemblages of the Permian Longtan Formation in the southeastern Sichuan Basin at Well X1 used core observations, optical thin-section observations, Ar-ion polishing, scanning electron microscopy and nitrogen adsorption tests to compare and analyze storage space types and pore structures in the shale to determine the sedimentary paleoenvironment, petromineralogy and organic content properties. The marine–continental transitional facies in the study area were deposited in a warm climate that favored enrichment by organic matter. The kerogen is type I (average vitrinite reflectance 2.66%), which is within the favorable thermal maturity range for the presence of shale gas. The lithology mainly comprises shale, siltstone and limestone (with bioclasts), and a coal seam. The lithological development divides the Longtan Formation into lower (swamp), middle (tidal flat/lagoon) and upper (delta) sub-members. From lower to upper divisions, the lithofacies evolved from silty shale to clay shale and then to

shale intercalated with siltstone or calcareous layers. The proportion of intergranular and dissolution pores in the clay minerals decreases gradually from lower to upper sub-members, and pore size sizes also tend to decrease. Relatively large diameter pores and microfractures occur in the inorganic matter in the lowest sub-member. Quartz and clay are the main constituents of the shale, respectively contributing to the specific surface area and specific pore volume of the reservoir space.

Keywords: Permian Longtan Formation; southeastern Sichuan Basin; lithological assemblages; shale reservoir characteristics

1. Introduction

Multi-cycle tectonic evolution and deposition in the Permian in China, several sets of organic-rich shale systems developed with marine, marine–continental transitional and continental facies [1]. Shale gas is abundant in the marine–continental transitional facies, amounting to approximately 19.8 × 10^{12} m^3, or 25% of the total shale gas reserves in China, and represents extensive exploration and development potential [2]. The presence of mainly Carboniferous–Permian marine–continental transitional shale gas had previously been confirmed in the Ordos, Sichuan, Bohai Bay and Qaidam Basins [3–5]. The Sichuan Basin underwent complex geological structural development from the Paleozoic to the Cenozoic, evolving from a craton basin to a foreland basin with complex superimposed basins containing both marine and continental facies [6, 7]. The source rocks in the Longtan Formation occur widely throughout the Sichuan Basin, mainly in the east, southeast and southwest. A large number of wells in the Sichuan Basin and the surrounding late Permian Longtan Formation have encountered varying amounts of gas in the shale, indicating favorable gas prospects [8–12]. The sedimentary facies of the Longtan Formation display obvious transformations from continental to marine facies (i.e., fluvial / swamp / tidal flat / shallow-water shelf / deep-water shelf)

located in an arc stretching from the southwest to the northeast [13, 14].

The Longtan Formation shale strata in the southeastern Sichuan Basin comprise both thin layers and large accumulated thicknesses containing a number of coal seams or coal lines, characteristically with frequently interbedded shale, coal, sandstone and limestone [15]. Transitional-facies reservoirs such as these differ from, for example, the Longmaxi Formation shale in terms of organic matter types and content. Marine–continental transition shale has attracted increasing attention in China in recent years, but relatively few studies have been reported on its reservoir-forming properties. Although the present comparative study of the pore structure of marine shale provides a certain understanding in key areas, comparative studies of the pore structure of shales in different sedimentary facies have yet to be carried out [16–20].

The lithological assemblages in the different thicknesses in the Longtan shale play a key role in hydrocarbon accumulation, storage and migration [8, 21–24]. The presence of many different lithologies and frequent depositional superposition, as well as complex lithofacies transformations, present a difficult obstacle to determining the mechanisms and main influences on shale gas enrichment, and therefore on the prediction of favorable exploitation areas [15]. To date, research on shale gas in the Longtan Formation has mainly focused on gas generation potential and gas bearing capacity [8, 25, 26] but few studies of reservoir development in different lithological assemblages are available; in particular, the exploration potential remains to be investigated [27]. Studies of the development of shale reservoirs in the Longtan Formation, especially the relationship between the different lithological assemblages, should be strengthened to provide a theoretical basis for gas exploration.

Numerous investigative methods and technologies were used in the present study: X-ray

diffractometry (XRD), organic geochemistry, organic maceral identification, Ar-ion polishing, scanning electron microscopy (SEM) and N_2 adsorption testing. In addition, comparisons and analyses of the different lithological assemblages and sedimentary properties of the Longtan Formation shale were conducted to define the properties of the reservoirs that developed over time in the different sedimentary environments described above. The findings lay a foundation for further exploration and development in the study area.

2. Materials and methods

2.1 ~~Samples~~

The Longtan Formation in the southern Sichuan Basin is a series of widely deposited coal-bearing rocks with a thickness of 20–120 m in parallel unconformable contact with the Maokou Formation. From land to sea, the sedimentary facies belt spreads roughly west–east and extends in a north–south direction, and mainly developed a tidal flat-lagoon sedimentary system [24, 28-30]. The water was relatively shallow, with a warm and humid tropical rainforest climate, and the development of peat swamps favored the growth, deposition and preservation of terrestrial plants, making it ideal for the enrichment of organic matter.

The samples were taken from Well X1 at the southeastern margin of the Sichuan Basin (Figure 1), located in the low-steep fold belt, southern Sichuan Basin. The lithology of the Longtan Formation is mainly black shale and carbonaceous shale, with coal seams developing [15].

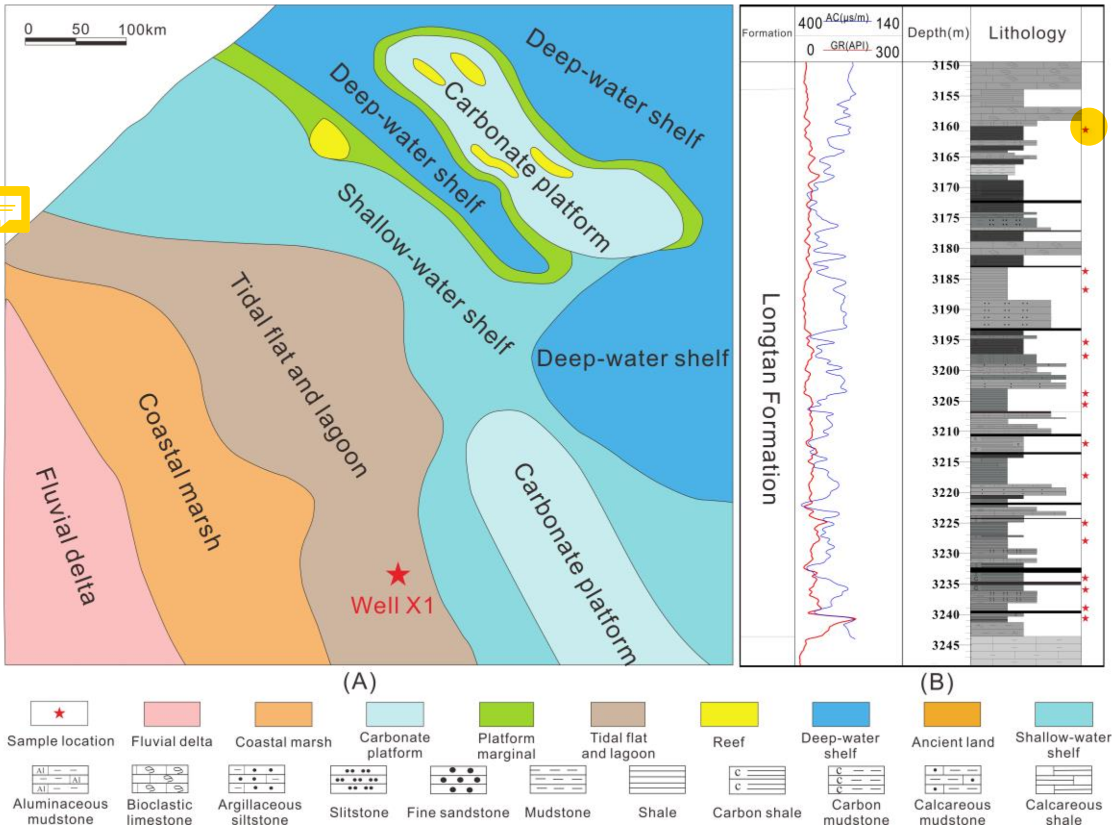

Figure 1. (a) Sample location; (b) Well X1 in Longtan Formation (modified from [29] and [30])

2.2. Methods

Total organic carbon (TOC) content was measured using a RJXWK-1 carbon-sulfur analyzer in accordance with the TOC test standard for sedimentary rocks (GB/T 19145-2003); The thermal evolution maturity (Ro) of the organic matter was measured using a Zeiss Axio Scope A1 polarized light microscope and TIDAS S MSP 400 spectrophotometer (J&M Analytik AG) in accordance with the standard vitrinite reflectance test procedure for sedimentary rocks (SY/T 5124-2012); Organic matter types were determined using a Zeiss Axio Scope A1 polarized light microscope in accordance with the standard method of kerogen maceral identification and classification (SY/T 5125-2014) [31]. The macerals, morphology and structure of kerogen were observed under transmitted light and fluorescence. Since the kerogen extraction process may destroy the structure of organic matter and the developmental state of some organic matter in shale and thereby affect the determination of the original source of the organic matter, the samples were observed in thin section.

A qualitative analysis of the mineral composition was conducted using a Zeiss Axio Scope A1 polarized light microscope in accordance with the thin section identification standard SY/T 5368-2016. A quantitative analysis of the mineral composition was carried out on the X'Pert Powder X-ray diffractometer (Malvern Panalytical) in accordance with the standard XRD analysis method for clay minerals and common non-clay minerals in sedimentary rocks, SY/T 5163-2010.

SEM testing was performed on a Zeiss Sigma 300 scanning electron microscope in accordance with the standard SEM procedure for rock samples (SY/T5162-2014), and the images were analyzed using image analysis software to determine the quantitative characterization of pore structure parameters (e.g., the number, diameter, width, perimeter and area) [32, 33].

Nitrogen adsorption testing determines the specific surface and pore size distribution of the

rock using the static nitrogen adsorption volume method (Micrometrics ASAP 2460 automatic specific surface and pore size analyzer) to complete the test process in accordance with GB/T 19587-2017/ISO 9277: 2010.

The content of major elements was determined using an AXios mAX X-ray fluorescence spectrometer (Malvern Panalytical) in accordance with standard GB/T 14506.28-2010). Testing accuracy was determined to be better than ± 3%.

3. Results

3.1. Lithological assemblages and sedimentary characteristics

In the study area, the carbonaceous shale contains some coal. From bottom to top, the evidence indicates that the Longtan Formation experienced a low energy in static water → medium-low energy → relatively low energy sequence [13, 34]. The lateral distribution of the formation in the southeastern Sichuan Basin was basically stable, with the strata thickness between 70 and 80 m, but the lithological content varies greatly within the different thicknesses [29]. The shale is mainly gray-black and black, with thin coal seams (perhaps better described as 'coal lines') and carbonaceous shale mixed with limestone, marlstone, silty-fine sandstone, coal lines and some siderite. Many such shale beds occur, all with numerous interbedded layers [15, 30, 35].

The mineral composition contents of fifteen samples from Well X1 were measured by XRD whole-rock and clay mineral tests (Figure 2). The differences in the lithological assemblages in showed that Longtan Formation could be further divided into the Lower, Middle and Upper Longtan Formations (Table 1).

The Upper formation is dominated by cross- and parallel-bedded deltaic sediments, and

containing plant fossils. Delta front subfacies are evident in this section. The lithology consists mainly of mudstone bioclasts, and shale mixed with limestone or siltstone, and occasional coal lines. The clay minerals predominate (average content **44.5%),** with about 33.1% carbonate minerals (calcite and dolomite), and quartz and feldspar clastics (about 18.00%).

The sedimentary environments of the Middle and Lower Longtan Formation are dominated by tidal flat and lagoon, and partly swamp, with wavy bedding, horizontal bedding and minor cross-bedding. Banded and nodular siderite occur together. All of these reflect low-energy sedimentary environments , relatively deep water, and a warm climate. The lithology of the Middle Longtan is mainly thick shale or carbonaceous mudstone, with a relatively thick coal seam. The average clay mineral content is 52.6%; the average content of clastic minerals (quartz and feldspar) is about 23.2% and the average carbonate minerals content is about 22.2%. Siderite is evident as nodules and powdery crystals.

Table 1. Comparison of lithological combinations in Permian Longtan Formation, Well X1, southeastern Sichuan Basin

Formation	Upper Longtan	Middle Longtan	Lower Longtan
Depositional model	Delta	Tidal flat and lagoon, developing swamp facies in the top	Tidal flat and lagoon, developing swamp facies in the bottom
Subfacies	Delta	Lagoon facies mainly, part tidal flat-swamp facies	Developing tidal flat- swamp facies, part of the lagoon facies
Microfacies	Peat swamp	Lagoon mud and intertidal mixed flat, peat swamp	Tidal flat-peat swamp, lagoon mud
Lithological combination	Limestone with bio-clastic, Siltstone with mud, Silty shale, Occasional thin coal seam	Thick shale with limestone and silty shale mainly, part of shale and sandstone interbedded, and developing coal seam and nodular siderite	Shale interbedded with siltstone and limestone mainly, developing thick carbonaceous shale and thick coal seam, and siderite distributed as granular and clumpy
Microscopic characteristics	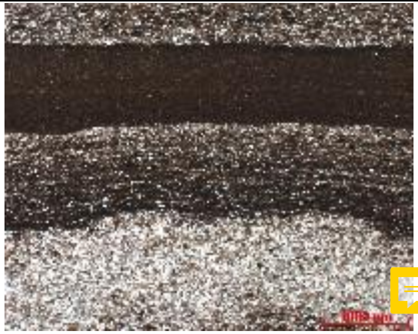	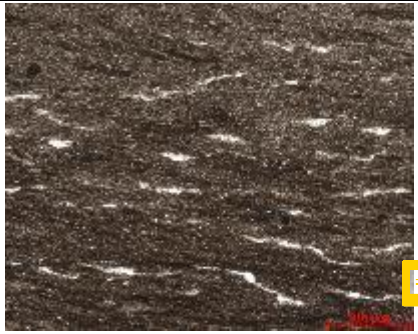	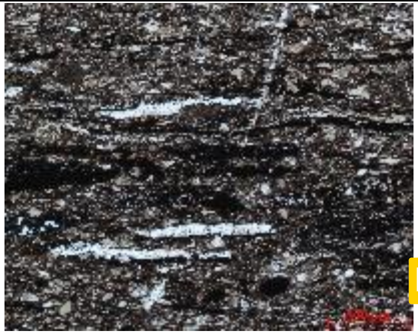
	Sample 3#: Argillaceous siltstone. The aggregation of clay minerals is mainly banded, and organic matter is clumpy and fine banded, 5×10 (-).	Sample 7#: Shale. Organic matter are distributed along the bedding, 5×10 (-).	Sample 9#: Silty shale. Silty lamination develops, and organic matter is black speckled, clumpy and banded, 5×10 (-).

	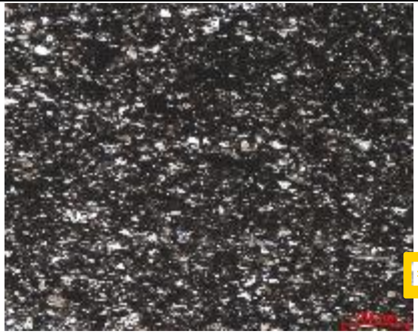	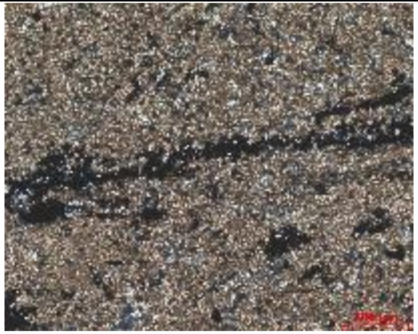	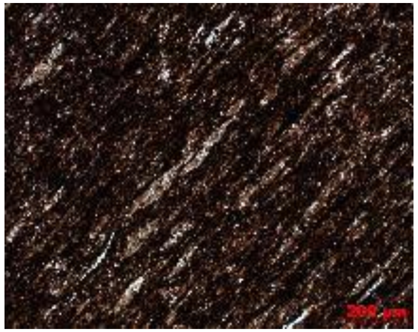
	Sample 3#: Siltstone with mud. Organic matter is black clumpy and banded, 5×10 (-).	Sample 4#: Shale with dolomite. Organic matter develops bedding, 10×10 (+).	Sample 6: Shale with siltstone. Bedding development, and organic matter develops bedding, 10×10 (-).
	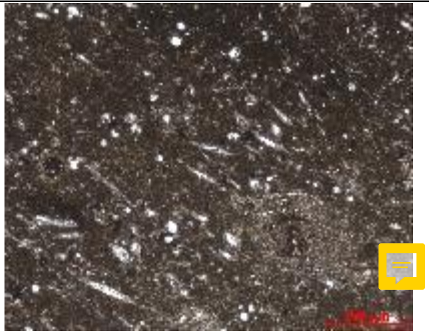	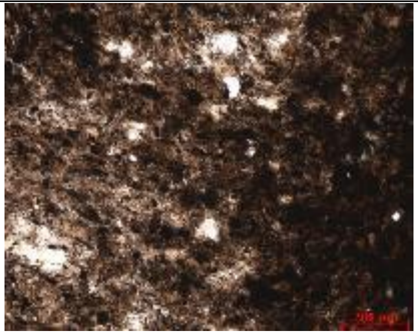	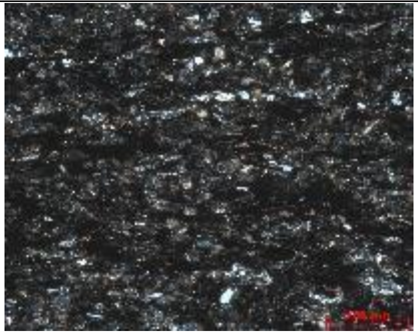
	Sample 3#: Shale with bioclasts. Bioclasts are mainly spongy spicules scattered in rocks in needle-like and elliptical shapes, 5×10 (+).	Sample 8#: Carbonaceous mudstone. The mudstone is yellow-brown in color and is mainly an aggregation of microcrystalline and cryptocrystalline clay minerals. A few cracks are filled with organic matter, 10×10 (-).	Sample 9#: Silty shale, 5×10 (+).

	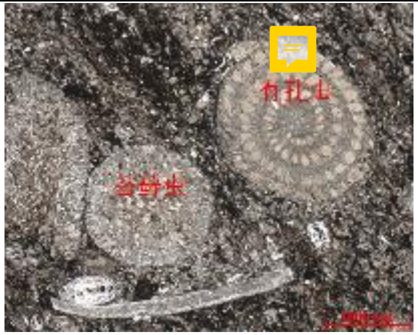	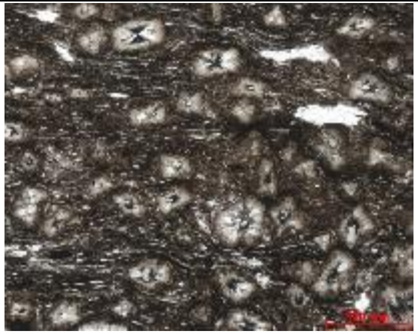	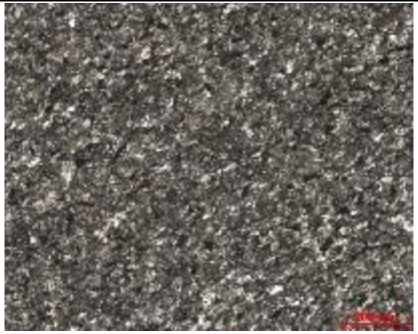
	Sample 14#: Bioclastic limestone. Bioclasts such as foraminifers and bryozoans were distributed elliptically, most of which were metasomatized by calcite and siliceous. A small number of ostracoda fragments were found scattered in the shape of tiny crescent, 5×10 (+).	Sample 6#: Mudstone. Siderite is nodular, spherical, and has a radial structure inside, with the overall characteristics of orthogonal extinction with sizes ranging from 0.1–1.0 mm, 10×10 (+).	Sample 9#: Argillaceous limestone. Siderite mainly consists of granular aggregates with fine microcrystals, and the whole aggregates are granular and clumpy, 5×10 (+).
Sedimentary structure	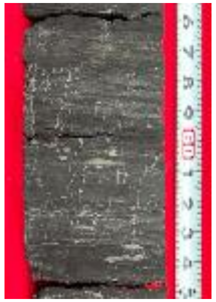	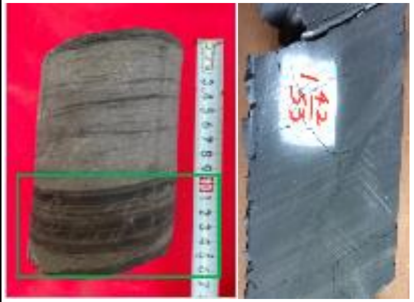	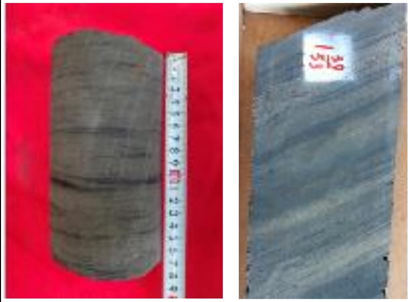
Bedding type	Developing bivalves and bryophytes Bioturbation, horizontal bedding, nodular siderite.	Horizontal bedding, wave-ripple bedding.	Lenticular bedding, veined bedding, cross bedding.

In the Lower Longtan, mudstone is mainly interbedded with siltstone and limestone, and thin coal seam is developed. The average content of clay minerals is 51.13%, and the average content of clastic minerals (quartz and feldspar) is 30.73%. The mean carbonate minerals content is 13.4%, mainly siderite, which is mostly spherical and modular. At the bottom of the Longtan Formation, the aluminaceous mudstone is developed and unconformably contacted with the limestone of the Maokou Formation.

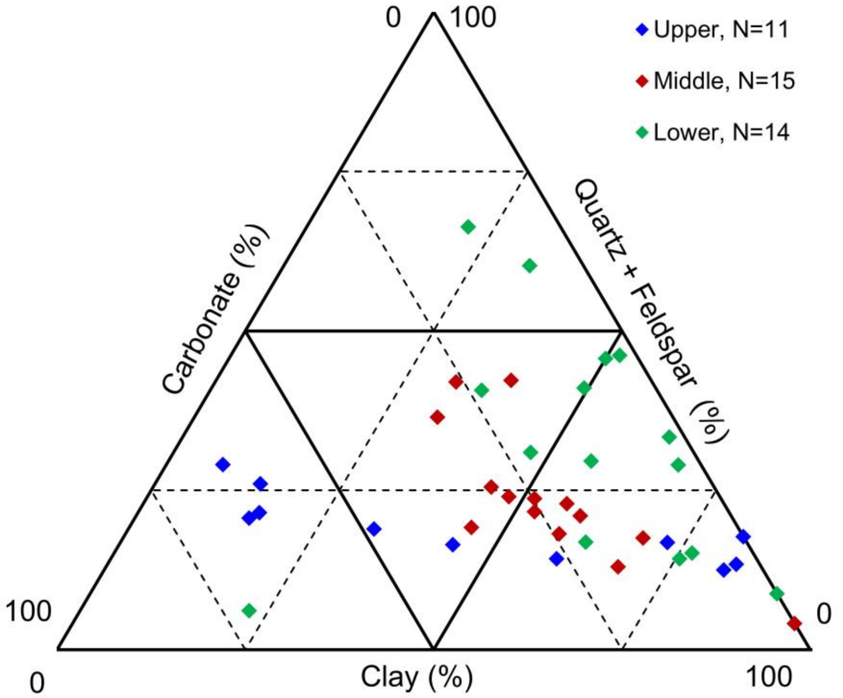

Figure 2. Triangulation of mineral composition of Permian Longtan Formation samples from southeastern Sichuan (Note: the datum is from this research and Sinopec Southwest Exploration and Development Research Institute)

3.2. Organic geochemistry characteristics

The type of organic matter not only determines the hydrocarbon generation capacity of source rocks and the properties of hydrocarbons, but also affects the occurrence and migration of gas in shale, and has an important influence on the development of different types of reservoir space. The maceral types of organic matter can be distinguished by the maceral morphologies obtained by

polarizing microscope. The analysis results (Table 2 and Figure 3) of organic matter types of eleven samples in Longtan Formation show that the kerogen types are mainly type III and type II_2-III, and the main macerals are exinite and vitrinite, with the mean content of 56.09% and 36.45% respectively, indicating that the organic matter is mainly formed by the fragments of reproductive organs and epidermal tissues of terrestrial higher plants after intense degradation, with the shape of clastic and/or partly flocculent. Occasionally, some vitrinite are attached with amorphous humus in around. Organic matter types in different lithology are different. Under a polarizing microscope, the maceral structure of different shale samples is given priority to with original structure, with massive structure and small scattered plant debris. The vitrinite with woody structure is commonly developed in coal samples, which is mostly banded or clumpy, suspected to be formed by the degradation of stems, leaves, lignocellulose or some marine lower plants.

The thermal maturity differences are little in the shale samples of Longtan Formation from the vitrinite reflectance test. The vitrinite reflectance ranges from 1.76% to 3.61%, with an average of 2.66%, and they are all in the high maturity stage.

Table 2. Analysis results of kerogen type of Longtan Formation samples (transmitted light + fluorescent)

Sample	Formation	Lithology	Sapropel (%)	Exinite (%)	Vitrinite (%)	Inertinite (%)	Type Index	Type
2#	Longtan Formation	Calcareous mudstone	-	66	28	6	6.00	II_2
3#	Longtan Formation	Carbonaceous mudstone	-	62	33	5	1.25	II_2
4#	Longtan Formation	Mudstone with limestone	-	42	53	5	-23.75	III
6#	Longtan Formation	Mudstone	-	54	41	5	-8.75	III
7#	Longtan Formation	Silty mudstone	-	54	41	5	-8.75	III
8#	Longtan Formation	Mudstone	-	23	71	6	-47.75	III

10#	Longtan Formation	Silty mudstone	-	64	32	4	4.00	II_2
11#	Longtan Formation	Carbonaceous mudstone	-	30	65	5	-38.75	III
13#	Longtan Formation	Argillaceous siltstone	-	66	30	4	6.50	II_2
14#	Longtan Formation	Shale	-	74	24	2	17.00	II_2
15#	Longtan Formation	Mudstone with siltstone	-	82	16	2	27.00	II_2

Note: "-" indicates that the contents are negligible.

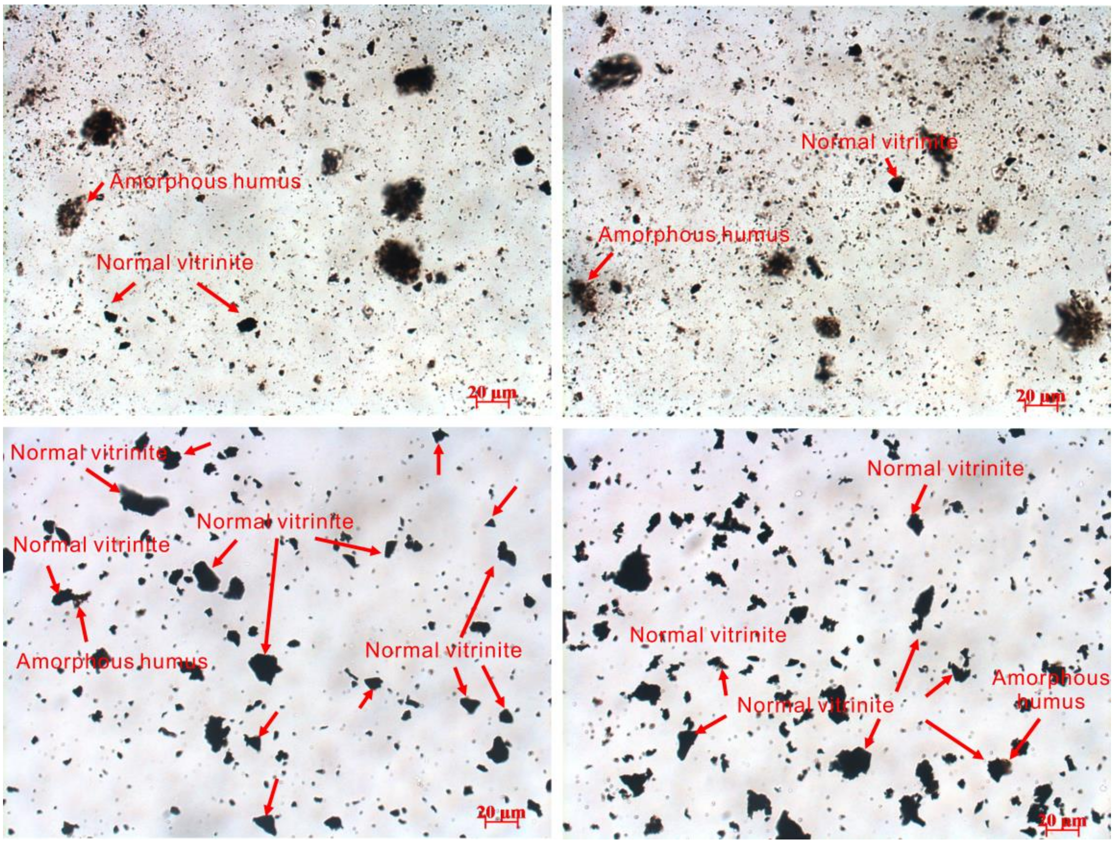

Figure 3. Organic photomicrographs of typical shale sample in Longtan Formation under fluorescent

The distribution characteristics of organic matter are affected by paleoclimate, paleoproductivity, water redox conditions and depositional rate. Chemical Index of Alteration (CIA)proposed by Nesbitt et al. (1982) is used to compare and analyze the paleoclimatic conditions during the shale deposition period of Permian Longtan Formation in well X1 [36], and the calculation formula is as follows:

$$\mathrm{CIA} = \left[\frac{Al_2O_3}{CaO^* + Al_2O_3 + Na_2O + K_2O}\right] \times 100$$

All oxide units are moles, and CaO* refers only to CaO in silicate minerals. In this paper, CaO* is calculated indirectly by P_2O_5, with the formula as following:

$$CaO^* = CaO - P_2O_5 \times 10/3$$

In generally, high CIA indicates the warm and wet paleoclimate, while low for dry and cold paleoclimate. In particular, when CIA is between 50 and 65, it reflects the dry and cold climate under the background of low chemical weathering. CIA between 65 and 85 reflects the warm and wet climate under the background of moderate chemical weathering, and CIA between 85 to 100 reflects the thermal and humid climate under the background of intensification weathering [36].

The CIA of different samples of the Longtan Formation in the study area range from 77.85 to 84.86, with an average of 81.62 (Table 3), and there is no significant difference between the CIA of delta environment and tidal flat-lagoon environment, indicating that the Longtan Formation shale was in a warm and wet climate during the deposition period. In addition, the rich of reduction authigenic minerals (siderite) in samples indicates a redox environment, which is conducive to the deposition of organic matter [37].

The TOC from the testing of fifteen samples of the Longtan Formation in three wells and collecting of thirty-nine data from references [13, 15, 30] indicates the TOC distribution of the Longtan Formation is wide and bimodal, ranging from 0.15% to 83.02% and mainly in 2%–4% and 10%–20%, with an average of 5.125% (Figure 4A). Meanwhile, the TOC of coal seam ranges from 20.49% to 83.020%. Because of the great varying of lithologies of the Longtan Formation, the TOC contents vary greatly in the vertical, showing strong heterogeneity. According to the test results and lithological assemblage classification results, the TOC distribution of different samples is

statistically analyzed. The TOC contents of twenty samples in the delta environment of the Upper Longtan formation range from 0.15% to 20.49%, with an average of 3.5%. The TOC contents of eleven samples in the tidal flat-lagoon environment of the Middle Longtan Formation range from 0.17% to 66.62%, with a mean

Table 3. Major element oxide contents of different samples of Longtan Formation in study area

Sample number \ Minimum detectable value	Major element oxide contents (%)										Ignition loss (%)	CIA(g/mol)
	SiO_2	Al_2O_3	MgO	Na_2O	K_2O	P_2O_5	TiO_2	CaO	TFe_2O_3	MnO		
	0.0335	0.0375	0.0277	0.0021	0.0382	0.0019	0.0333	0.0438	0.0078	0.0138	/	83.97
1#	40.51	13.47	2.26	0.52	1.84	0.17	2.60	11.52	8.02	0.10	18.34	84.01
2#	33.57	14.16	2.89	0.74	0.91	0.31	2.83	7.73	15.89	0.15	18.01	79.60
3#	42.62	24.25	0.83	1.07	1.34	0.33	4.57	0.70	6.60	0.02	17.37	84.86
4#	38.04	23.34	1.02	1.10	0.90	0.21	5.08	0.75	12.43	0.17	16.62	83.78
5#	43.35	24.07	1.33	1.10	0.96	0.36	4.57	1.58	7.53	0.06	14.22	80.87
6#	40.30	23.19	1.15	1.45	1.40	0.40	4.79	0.86	9.58	0.11	16.32	81.94
7#	41.71	15.07	3.43	0.63	0.48	0.37	4.20	5.46	16.22	0.09	9.81	81.20
8#	35.27	10.01	3.83	0.15	0.20	0.43	1.70	11.80	19.84	0.16	15.00	81.72
9#	40.64	19.15	2.12	1.38	1.32	0.42	3.11	4.12	11.90	0.16	14.80	83.56
10#	36.34	12.73	2.61	0.59	0.52	0.18	1.59	1.08	28.45	0.30	15.81	77.85
11#	40.92	19.39	1.74	1.14	1.63	0.41	3.66	2.48	12.28	0.16	15.44	79.90
12#	38.92	23.07	1.09	0.87	1.97	0.06	4.10	0.62	14.45	0.04	14.47	82.84
13#	39.58	22.98	0.66	1.17	1.46	0.21	3.78	0.69	10.15	0.20	19.53	79.69
14#	41.36	25.39	0.47	0.62	0.83	0.23	4.55	0.50	6.06	0.11	20.11	79.00
15#	37.94	19.62	1.35	0.59	3.02	0.45	3.36	3.51	14.37	0.15	13.11	81.10

of 4.01%. The TOC contents of twenty-three samples in the tidal flat-lagoon environment of the Lower Longtan Formation range from 0.42% to 83.02%, with a mean of 9.37%. The variation of TOC content in lithological assemblages of different sedimentary environments is complex, with bimodal characteristics. The TOC peak values (TOC > 20%) of samples in the tidal flat-lagoon of the Middle and Lower Longtan Formation occurs in coal and carbonaceous mudstone, and TOC contents are relatively low in argillaceous limestone and siltstone with mud of the Upper Longtan Formation (Figure 4B).

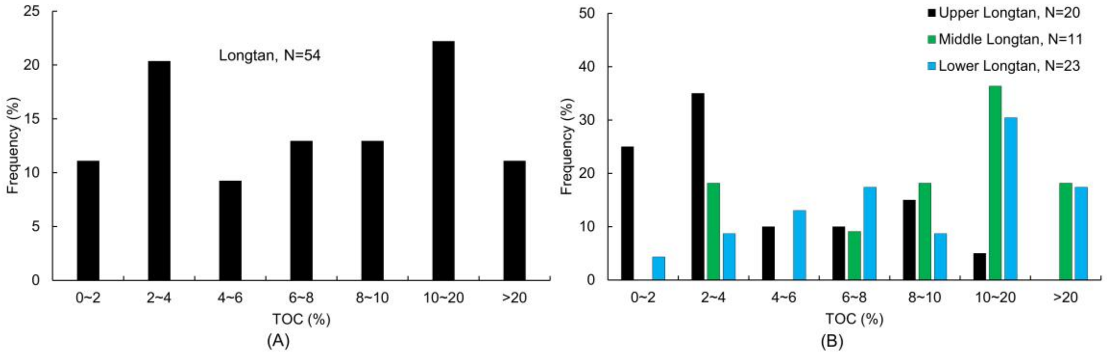

Figure 4. The distribution of TOC contents of Longtan Formation samples (the datum are from this research and reference [13, 15, 30])

3.3. Development characteristics of reservoir space

3.3.1. Pore types

Ar-ion polishing and field emission SEM were used to observe the development characteristics of samples' microscopic storage space of the Longtan Formation in the study area. Based on the pore types classification of organic-rich shale [38] and the genetic and structural characteristics of shale pores, inorganic pores, organic pores and microfractures are identified as the main storage space of marine-continental transitional shale in Longtan Formation.

Inorganic pores are mostly distributed among clay mineral particles or in clastic particles such as quartz. Chemically unstable clay minerals (montmorillonite) will produce a large number of pores

in the process of deposition and burial, and transform into andreattite or illite. A large number of interlayer pores and fractures develop in the lamellar andreattite aggregation, showing as curved (Figure 5A). The interbeded and intrastratal micropores not only create space for shale gas occurrence, but also provide microscopic migration channels for gas seepage. Some minerals (quartz and pyrite) are interfered by the external environment during the self-growth process, resulting in the formation of intragranular pores in the crystal accumulation process (Figure 5B). The pore size varies from several nanometers to several-hundred nanometers, with relatively low development degree and poor the connectivity. Occasionally, intercrystal (intergranular) dissolution pores occur at the edge of siderite due to dissolution (Figure 5C), with the shape of bays or long-narrow. Few dissolution pores are observed relatively under microscopic observation in the study area. Under microscope, the occurrence patterns of organic matter in Longtan Formation can be filled, dispersed and bedding enrichment. Some organic matters have certain structural characteristics, but with low organic matter pores, and some of them are isolated circular or oval (Figure 5D, E and F). Microfractures are mainly developed in the intercrystal or interparticles of clay mineral, and little at the edge of organic matter. They are mainly exogenous cracks and intergranular fractures formed under the action of external forces, with the width of 1-20 μm generally. Some microfractures cut through the particles of clasolite or clay mineral straightly (Figure 5G and H), and some shrinkage fractures are also observed between organic matter and mineral particles (Figure 5I), mainly due to volume shrinkage of organic matter during thermal evolution. The results show that the dominant storage spaces in different samples are pore-fractures and micro-fractures between clay mineral, and the organic pores are poor developed.

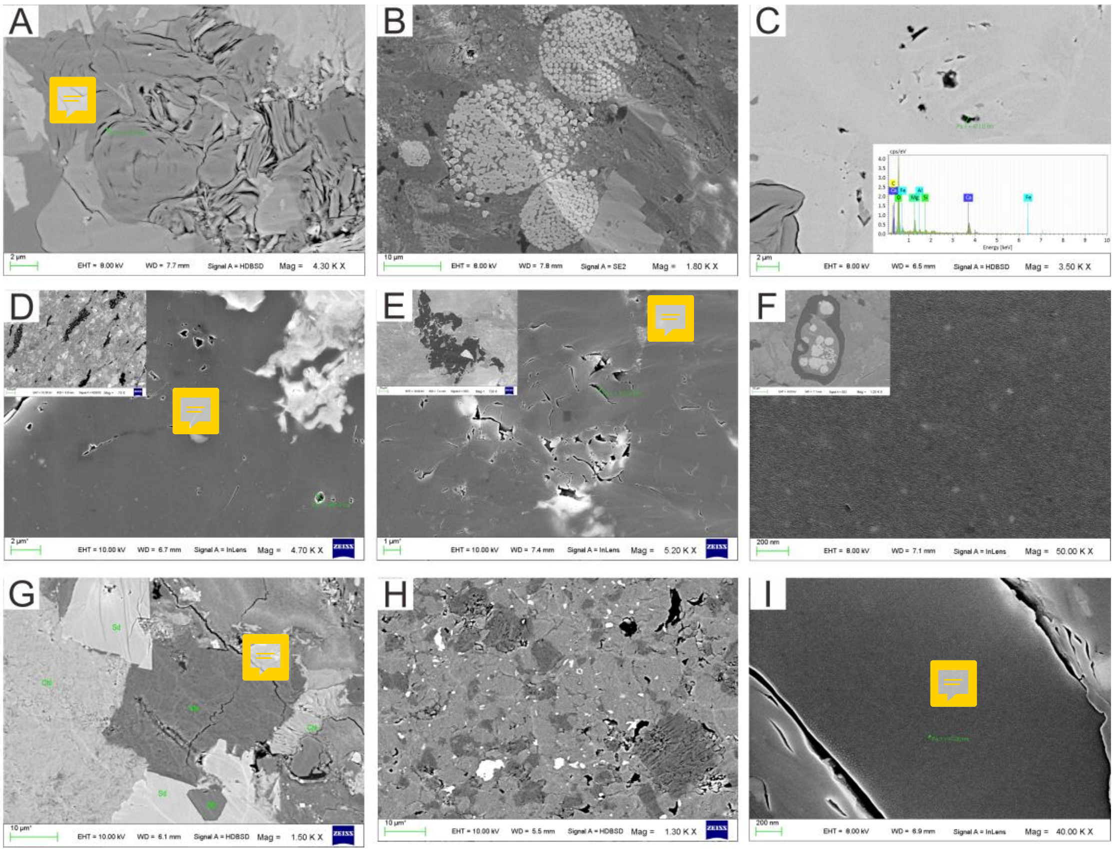

Figure 5. Distribution characteristics of microscopic reservoir space in different shale samples of the Longtan Formation

A. Sample 8#: interlayer fractures developing in lamellar clay minerals (×4300); B. Sample 2#: Framboidal pyrite aggregations with parts of the intercrystal pore are filled with clay minerals (×1800); C. Sample 5#: Irregular dissolution pores in siderite (confirmed by energy spectrum) (×3500); D. Sample 9#: The sample structure is tight, clastic interbedded with clay minerals and organic matter, and the organic matter is fragmentary or interstitial (×70). Irregular pores and microfractures are developed in fragmentary organic matter (×4700); E. Sample 12#: Interstitial organic matter interbedded with clay minerals and siderite, and developing pores and fractures (×5200); F. Sample 4#: The interior of crust organic matter is filled with micro-granular and framboidal pyrite, and clay minerals (×1200) with little pores (×50000); G. Sample 9: Microfractures (×1500); H. Sample 12#: Micro-granular quartz in inlaying contact interbedded

with lamellar kaolinite aggregations; intergranular pores and fractures develop (×1300); I. Sample 14: The pores in banded organic matter are not developed, but shrinkage fractures develop at the edge of organic matters (×40000).

3.3.2. Development characteristics of pore structures

Porosities and pore structures developing characteristics of different samples are measured by Helium gas method and low-temperature N_2 adsorption. BET model and BJH model are used to calculate the specific surface area and specific pore volume, respectively. From the testing results, the porosities are ranging from 1.07% to 8.17%, mainly in 2%–4% and 6%–8% (Figure 6). Specific surface areas are mainly distributed from 1.122 m^2/g to 24.452 m^2/g, with an average of 11.619 m^2/g, and specific pore volumes rang from 0.001 mL/g to 0.032 mL/g, with a mean of 0.016 mL/g.

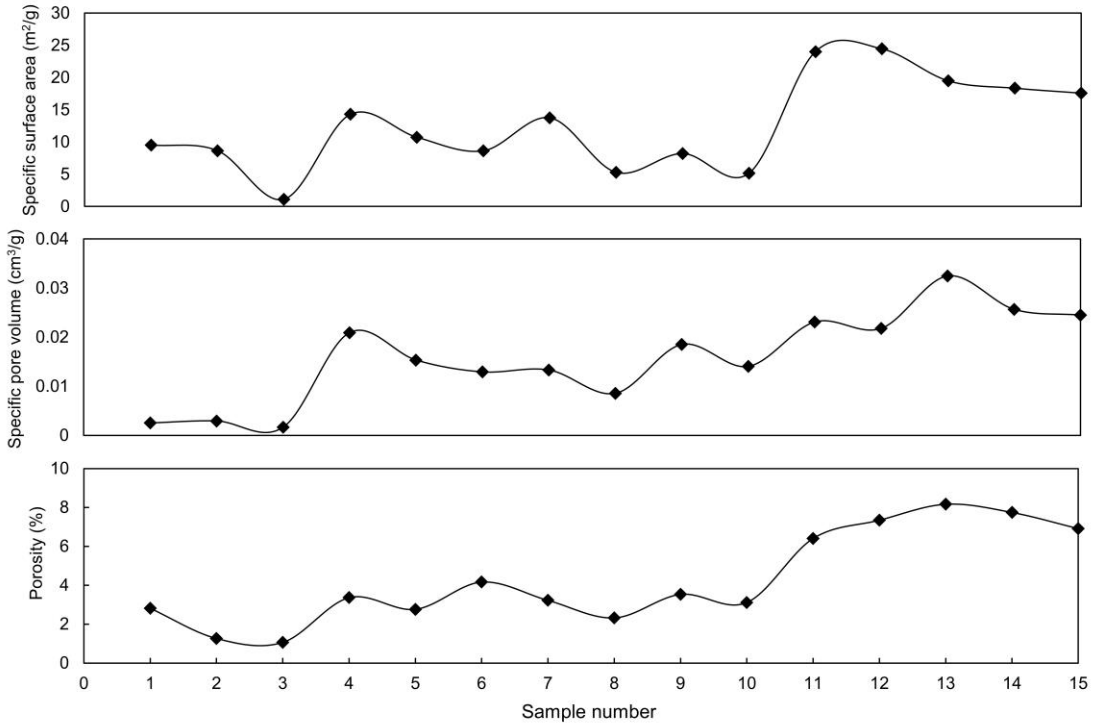

Figure 6. The distributions of porosity, specific surface area and specific pore volume of different samples

The morphological characteristics of hysteresis curve of isothermal adsorption-desorption

curve can reflect the morphological characteristics of pores qualitatively to some extent. However, the pore types of shale reservoir are diverse, and the hysteresis curve is often the superposition of various typical curves. According to the classification of nitrogen adsorption and desorption isothermal curve of IUPAC, the morphology of sample adsorption loop can be divided into three categories. The first adsorption loop (type I, represented by sample 13#) has both curve characteristics of type H2 and type H3 of IUPAC. Adsorption and desorption curves basically coincide in the low pressure section ($0 \leq P/P_0 < 0.4$). The obvious inflection point and wide hysteresis loop appear in the middle pressure section ($0.4 \leq P/P_0 < 0.8$), indicating the complex pore structure and multiple pore types, and characterized by ink-bottle types pores. The slope of the adsorption and desorption curves increases in the high pressure section ($0.8 \leq P/P_0 < 1.0$), indicating the development of fluted open pores in macropore. The morphology of second adsorption loop (type II, represented by sample 9#) is similar to type I adsorption loop, but the difference is that the slopes of adsorption and desorption curve don’t increase significantly in the high pressure section, indicating that the number of fluted open pores is less, mainly ink-bottle type pores. The third adsorption loop (type III, represented by sample 3#) is similar to pores of type H4 of IUPAC. The adsorption/desorption curves in low-pressure and medium-pressure sections almost overlap, and the inflection point is not obvious, indicating that the pores are single and mostly slit micropores.

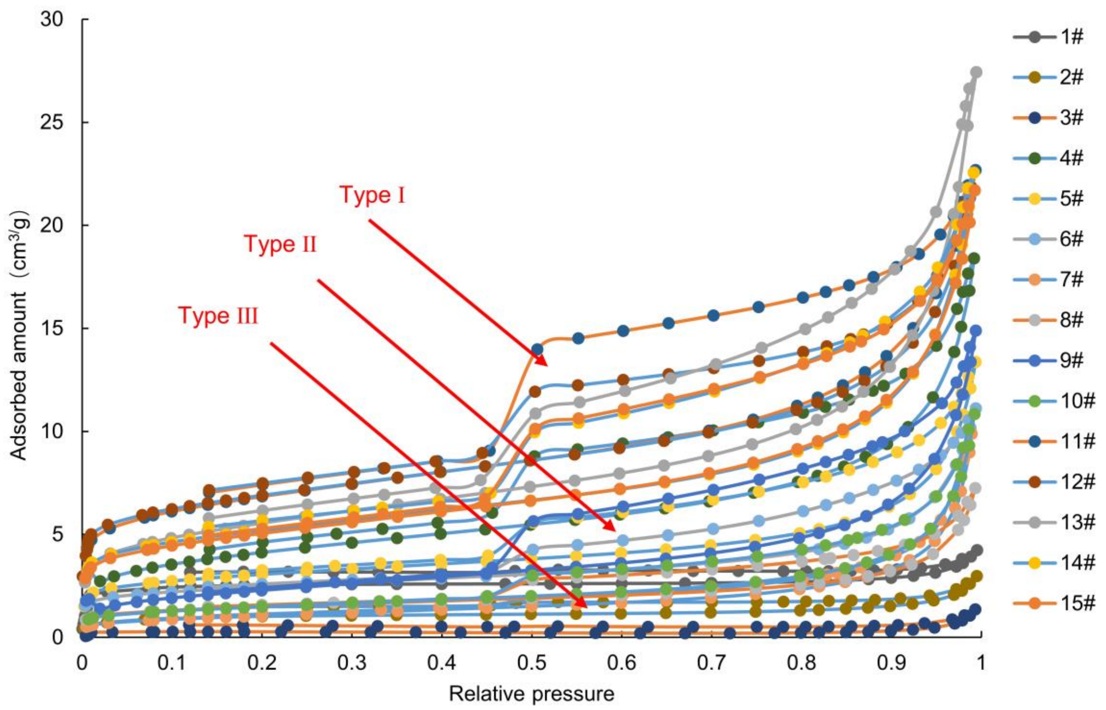

Figure 7. Isotherm adsorption and desorption curves of low temperature nitrogen of different samples

4. Discussion

The microscopic pore types, occurrence state and enrichment degree of shale gas are controlled by sedimentary facies, TOC contents, mineral compositions and tectonic evolution [1, 34, 39]. The sedimentary environment is one of the external factors controlling the formation of reservoir storage space, while the lithology and mineral composition controlled by the sedimentary environment are the internal factors controlling pore development. Therefore, the control effect of different lithological assemblages on shale pores is of great significance [40-42]. Image analysis software is used to identify the number, size and face ratio of pores in different shale samples of the Longtan Formation [32], and combined with the results of N_2 adsorption and desorption test, the distribution of pore structure characteristic parameters in different samples is significantly different. In the samples of mudstone with silty and calcareous mudstone from the Upper Longtan Formation, the

number of pore is relatively few, mainly inorganic pores, with pore size lower than 20 nm (Figure 8A and B). The specific surface area and pore volume of the samples are relatively small (Figure 6) , which are mainly micropores. In the thick organic-rich shale samples of the Middle and Lower Longtan Formation, organic matter is only distributed sporadically among detrital particles, and isolated high round organic pores are developed (Figure 8), with relatively small pore size. Inorganic pores are mainly interlayer pores of clay minerals, and occasionally developed a few mineral grain margin fractures. The pore size is mainly micropore and mesopore, presenting a bimodal, with the peak values at about 10 nm and 50 nm, respectively (Figure 8B and E). Some micron storage spaces are occasionally developed. Micropore is the main contributor to specific surface area, mesopores and macropores are the main contributor to pore volume. In the shale samples with organic matter from the sandstone-mudstone interbedded of the Middle and Lower Longtan Formation, inorganic pores and microfractures are relatively well developed, but poor for organic pores relatively. Organic matter is distributed in clumps between clastic particles or clay minerals, and some organic pores are connected. The specific surface area of the organic pores can provide specific surface for the adsorption of hydrocarbons. Inorganic pores include intercrystal pore of clay minerals, interparticle pore of quartz and feldspar, and dissolution pore of carbonate minerals. Silt particles are more line contact. Clay minerals mostly occur between silt particles, forming a large number of interlayer pores of clay minerals with large pore volumes (Figure 8), and providing good storage space for free hydrocarbons. Microfractures are mainly shown as mineral shrinkage and dissolution pore-fractures caused by diagenesis (Figure 8). The microfractures provide space for shale gas storage and can also serve as channels for shale gas migration. The pore size distribution generally shows the characteristics of multi-peak, with the peak values at 5 nm, 50–150 nm and 1–5 μm respectively

(Figure 8C and F). Figure 8 shows that the specific surface area of organic-rich shale samples in the Middle and Lower Longtan Formation is mostly contributed by micropores, and mesopores and mesopores make the largest contribution to pore volume.

From bottom to top, the organic pores in the organic-rich shale of Longtan Formation are poor as a whole, and the storage space is mainly inorganic pores and microfractures. With the evolution of shale lithofacies from silty shale to clay shale and then to shale with silty or calcareous shale, the proportion of interparticle pores of clay minerals and dissolution pores decreases gradually, and the pore size decreases as well. In the silty shale of the Lower Longtan Formation, inorganic pores and microfractures are relatively developed with large pore size (Figure 8).

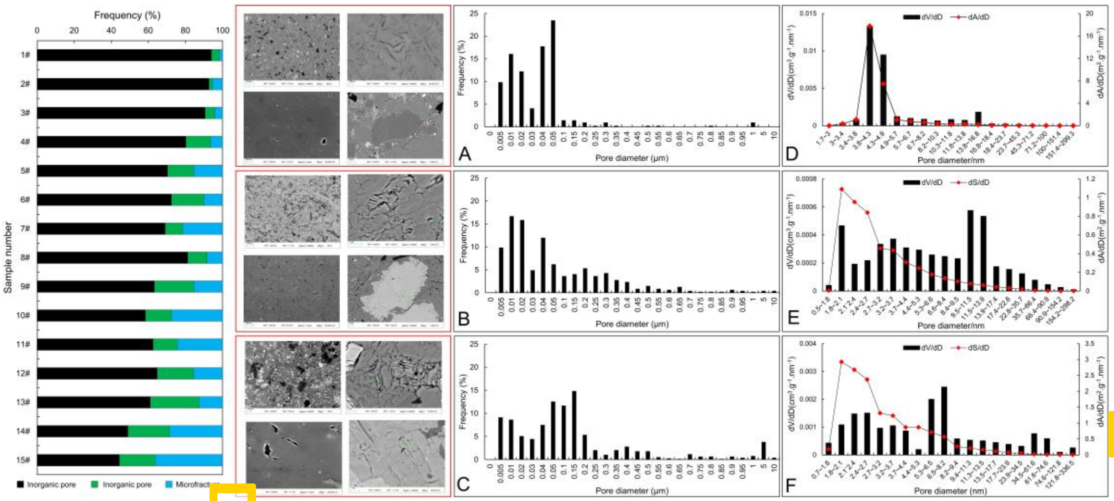

Figure 8. Pore types and structure characteristic parameters in different shale by SEM and image analysis

The development of reservoir storage space is controlled by different mineral compositions in shale reservoir. Quartz and clay minerals, as the main components of shale, have different influence degrees on the development of storage space [10]. Statistics found that the quartz contents of different shale samples from Longtan Formation in the study area have low correlation with the specific surface area (Figure 9A), and positively correlation with specific pore volume (Figure 9B).

Clay mineral was positively correlation with specific surface area of different samples (Figure 9D), but not obvious with specific pore volume (Figure 9E). The reason why is brittle minerals such as quartz in the samples from marine-continental transitional sedimentary environment often show good compaction resistance, which makes the rocks preserve some primary pores under compaction (Figure 9Fand G) [43]. The brittleness index (BI) of different samples are calculated based on mineral composition. BI = (quartz / (quartz + clay minerals + calcareous minerals))×100. The results show that the proportion of microfractures in all storage spaces increases with the increase of BI in different samples (Figure 9C), and the presence of quartz plays a positive role in the development of microfractures. Clay minerals are mainly chlorite and andreattite. Lamellar silicate minerals are often found in lamellar andreattite and chlorite aggregate with interlayer pores and large surface due to the fine particles (Figure 9H and I).

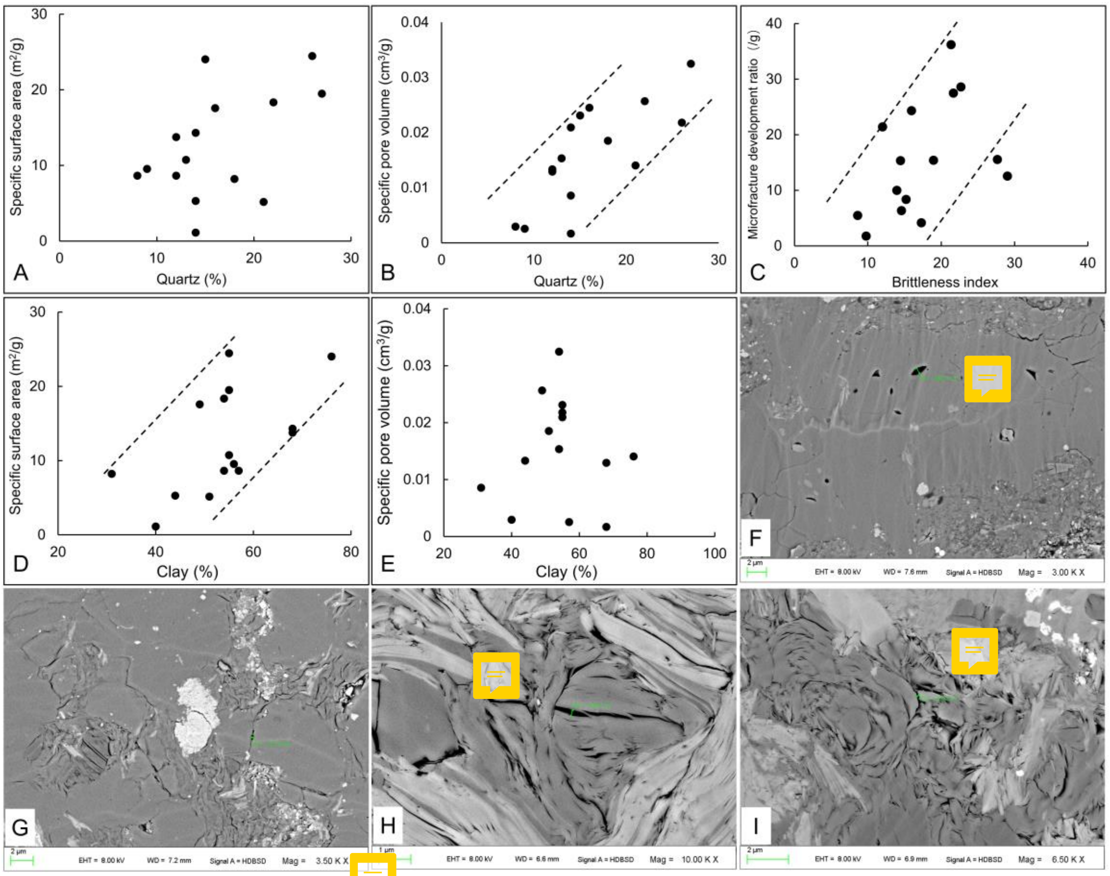

Figure 9. The relationship between the different mineral components (quartz, clay minerals) and

pore volume, specific surface area and microfracture development ratio

A. The relationship between the quartz and specific surface area; B. The relationship between the quartz and specific pore volume; C. Correlation between microfracture development ratio and brittleness index; D. The relationship between the clay minerals and specific surface area; E. The relationship between the clay minerals and specific pore volume; F. An irregular pore and microcrack in microgranular quartz (×3000); G. Particle margin fractures in microcrystalline quartz (siliceous) (×3500); H. Interbedded fracture in lamellar andreattite aggregation (×10000); I. Interlayer pores in alternately distributed lamellar andreattite and chlorite aggregation (×6500)

In the Middle and Lower Longtan Formation, the lithological assemblage is sandstone, mudstone, limestone interbedded with coal seam. Coal seam, as organic aggregation, has much higher gas generation capacity than shale, and coal gas will migrate to the adjacent siltstone and shale to accumulate and increase gas bearing capacity [10]. In addition, the high content of brittle minerals is conducive to the generation of natural or induced fractures (Figure 9G and H), which is conducive to the desorption, seepage, accumulation and exploitation of natural gas adsorbed in shale as well. One of the main forms of shale gas is the adsorption of clay minerals. As a good carrier of shale gas migration and accumulation, clay minerals also play an important role in the process of shale gas occurrence, migration and accumulation. Part of the organic matter is distributed among clay mineral grains under microscope, and adsorbing the gas molecules during shale gas generation. Illite and andreattite in clay minerals can promote kerogen cracking and play a catalytic role in organic gas generation. In addition to the high content of brittle minerals, organic-rich shale in the lithological assemblage of high frequency interbedded of sand-mud-coal has developed fissile and lamination, and the large lithological difference between layers causes the stress weak surface

increasing, and a large number of horizontal fractures are easily formed under the action of external forces, which promotes the radial extension of hydraulic fractures.

6. Conclusions

(1) The Longtan Formation in the study area belongs to the marine-continental transitional facies with complex lithology, mainly developed delta and tidal flat-lagoon sedimentary system. Combined with the characteristics of different lithological assemblages in the marine-continental transitional sedimentary environment, the Longtan Formation is further divided into the Lower Longtan Formation, Middle Longtan Formation and Upper Longtan Formation. The Upper Longtan Formation is dominated by deltaic sedimentation, with cross bedding and parallel bedding, and plant fossils develops. The sedimentary environment of the Middle and Lower Longtan Formation is dominated by tidal flat and lagoon, and partly swamp, with wavy bedding, horizontal bedding and small cross bedding, and banded siderite and nodular siderite are developed together. The main lithology includes thick shale interbedded with limestone, siltstone and interbedded shale, siltstone and limestone, with thick coal seam.

(2) The kerogen types of different lithological assemblages in shale are type III and type II_2-III, with the mean vitrinite reflectance of 2.66%, indicating the favorable thermal maturity for shale gas exploration. Inorganic pores are the main storage space of shale reservoir, with a few organic pores and microfractures. The difference of hysteresis loop in the low-pressure N2 adsorption and desorption test results indicate that three different types of storage space are developed.

(3) The storage characteristics of shale reservoirs in different lithological assemblages are compared and analyzed. The organic pores of organic-rich shale in Longtan Formation are not well developed as a whole, and the types of storage space are mainly inorganic pores and micro-fractures.

With the evolution of shale lithofacies from silty shale to clay shale and then to shale with silty or calcareous shale, the proportion of interparticle pores of clay minerals and dissolution pores decreases gradually, and the pore size decreases as well. In the silty shale of the Lower Longtan Formation, inorganic pores and microfractures are relatively developed with large pore size. Clay minerals play a positive role in the specific surface area of the sample, while quartz positive for the protection of the specific pore volume.

References

1. Zhao, P.R.; Gao, B.; Guo, Z.F.; Wei, Z.H., Exploration potential of marine－continental transitional and deep-water shelf shale gas in Upper Permian，Sichuan Basin. *Petroleum Geology & Experiment* **2020**, 42, (03), 335-344.
2. Dong, D.Z.; Wang, Y.M.; Li, X.J.; Zou, C.N.; Guan, Q.Z.; Zhang, C.C.; Huang, J.L.; Wang, S.F.; Wang, H.Y.; Liu, H.L.; Bai, W.H.; Liang, F.; Lin, W.; Zhao, Q.; Liu, D.X.; Qiu, Z., Breakthrough and prospect of shale gas exploration and development in China. *Natural Gas Industry* **2016**, 36, (01), 19-32.
3. Dong, D.Z.; Qiu, Z.; Zhang, L.F.; Li, S.X.; Zhang, Q.; Li, X.T.; Zhang, S.R.; Liu, H.L.; Wang, Y.M., Progress on sedimentology of transitional facies shales and new discoveries of shale gas. *Acta Sedimentologica Sinica* **2021**, 39, (01), 29-45.
4. Zuo, X.C.; Li, C.L.; Zhang, J.L.; Ma, G.Y.; Chen, P.P., Geochemical characteristics and depositional environment of the Shahejie Formation in the Binnan Oilfield, China. *J Geophys Eng* **2020**, 17, (3), 539-551.
5. Yuan, Y.Y.; Li, Z.P.; Zhong, M.Y.; Wang, Y., Pore structure characteristics of Taiyuan Formation coal measures shale in south central Qinshui Basin. *Coal Science and Technology* **2021**, 49, (09), 184-192.
6. Li, H.K.; Li, Z.Q.; Long, W.; Wan, S.S.; Ding, X.; Wang, S.Z.; Wang, Q.Z., Vertical configuration of Sichuan Basin and its superimposed characteristics of the prototype basin. *Journal of Chengdu University of Technology (Science & Technology Edition)* **2019**, 46, (03), 257-267.
7. Potter, C.J., Paleozoic shale gas resources in the Sichuan Basin, China. *Aapg Bull* **2018**, 102, (06),

987-1009.

8. Xiao, M.G. Sequence Stratigraphy and Geochemistry of Upper Permian Longtan Formation in Huayingshan，Northeast Sichuan Basin. Doctor, China University of Geosciences (Wuhan), Wuhan, 2012.
9. Ma, Y.S.; Cai, X.Y., Exploration achievements and prospects of the Permian-Triassic natural gas in northeastern Sichuan basin. *Oil ＆ Gas Geology* **2006**, 27, (06), 741-750.
10. Zhang, X.Q.; Li, X.Q.; Li, Y.Y.; He, Y.K.; Zhang, J.Z.; Zhang, Y.C., Ｒesearch progress of reservoir of shale gas in coal measures. *Coal Geology of China* **2020**, 32, (02), 59-66.
11. Xie X.; Hu L.; Deng H.; Gao J., Evolution of pore structure and fractal characteristics of marine shale during electromagnetic radiation[J]. PLoS ONE, 2020, 15(10):e0239662.
12. Chen F.; Lu S.; Xue D.; He X., Shale gas reservoir characterization: A typical case in the Southeast Chongqing of Sichuan Basin, China[J]. PLoS ONE, 2018, 13(6):e0199283.
13. He, Z.L.; Nie, H.K.; Li, S.J.; Liu, G.X.; Ding, J.H.; Bian, R.K.; Lu, Z.Y., Differential occurence of shale gas in the Permian Longtan Formation of Upper Yangtze region constrained by plate tectonics in the Tethyan domain. *Oil ＆ Gas Geology* **2021**, 42, (01), 1-15.
14. Cao, Q.G.; Liu, G.X.; Zhang, Z.J.; Pan, W.L., Sedimentary environment and its controlling on source rocks during late Permian in Sichuan Basin. *Petroleum Geology ＆ Experiment* **2013**, 35, (01), 36-41.
15. He, Y.; Tang, X.; Dan, Y.S.; Liu, G.X.; Xie, H.X.; Ma, Z.J., Lithofacies division and comparison and characteristics of Longtan Formation shale in typical areas of Sichuan Basin and its surrounding. *Natural Gas Geoscience* **2021**, 32, (02), 174-190.
16. Timms, N.E.; Olierook, H.K.H.; Wilson, M.E.J.; Delle Piane, C.; Hamilton, P.J.; Cope, P.;

Stütenbecker, L., Sedimentary facies analysis, mineralogy and diagenesis of the Mesozoic aquifers of the central Perth Basin, Western Australia. *Mar Petrol Geol* **2015**, 60, 54-78.

17. Jia, Y.Q.; Liu, Z.P.; Ren, X.H.; Zhou, Y.B.; Zheng, A.L.; Zhang, J.; Han, D.L., Organic Matter Type Differentiation Process and Main Control Mechanism: Case Study of the Silurian Longmaxi Formation Shale Reservoir in Weiyuan Area. *Acta Sedimentologica Sinica* **2021**, 39, (02), 341-352.
18. Pang, M.; Chen, H.X.; Tang, H.M.; Feng, Y.T.; Fang, T.; Liu, Y.G.; Zhao, S.C.; Wang, Y.F., Differences of micropore structure between marine shale and continental shale: Examples from Longmaxi Formation in southern Sichuan Basin and Yanchang Formation in Ordos Basin. *Natural Gas Exploration and Development* **2018**, 41, (02), 29-36.
19. Shu, Y.; Xu, Y.; Jiang, S.; Zhang, L.; Zhao, X.; Pan, Z.; Blach, T.P.; Sun, L.; Bai, L.; Hu, Q.; Sun, M., Effect of Particle Size on Pore Characteristics of Organic-Rich Shales: Investigations from Small-Angle Neutron Scattering (SANS) and Fluid Intrusion Techniques. *Energies* **2020**, 13, (22), 6049.
20. Wang, S.; Wang, J.; Zhang, Y.; Li, D.; Jiao, W.; Wang, J.; Lei, Z.; Yu, Z.; Zha, X.; Tan, X., Relationship between Organic Geochemistry and Reservoir Characteristics of the Wufeng-Longmaxi Formation Shale in Southeastern Chongqing, SW China. *Energies* **2021**, 14, (20), 6716.
21. Zhao, R.Q.; Chong, X.; Zhao, D.F.; Guo, Y.H., Longtan Formation Transitional Facies Coal Measures Shale Reservoir Development Features in Caohu Area. *Coal Geology of China* **2018**, 30, (10), 39-42+69.
22. Liu, S.X. Reservoir Characteristics and Sedimentary Control Mechanism of the Marine-Continental Transitional Mud Shale: A case of the Longtan Formation in Zhina Coalfield. Doctor, China University of Mining and Technology, Xuzhou, 2018.

23. Liu, C.Q.; Guo, S.B., Ｒeservoir characteristics of high clay content and microporous tight litharenites in marine—continental transitional environments. *Petroleum Geology ＆ Experiment* **2020**, 42, (02), 223-232.

24. Zhang, J.Z.; Li, X.Q.; Guo, M.; Dong, Z.L.; Wang, Z.; Fu, Q.H.; Wang, F.Y., Microscopic Pore Characteristics and Its Influence Factors of the Permian Longtan Formation Shales in the Southern Sichuan Basin. *Natural Gas Geoscience* **2015**, 26, (08), 1571-1578.

25. Zhang, J.Z.; Li, X.Q.; Wang, Y.; Fu, Q.H.; Cai, Y.Q.; Niu, H.Y., Accumulation conditions and reservoir characteristics of marineterrigenous facies coal measures shale gas from Longtan Formation in South Sichuan Basin. *Journal of China Coal Society* **2015**, 40, (08), 1871-1878.

26. Chen, Z.Q., Exploration for Shale Gas of Longtan Member in Permian Leping Formation, Sichuan Basi. *Natural Gas Technology and Economy* **2011**, 5, (02), 21-26+78.

27. Bao, S.J.; Lin, T.; Nie, H.K.; Ren, S.M., Preliminary study of the transitional facies shale gas reservoir characteristics: Taking Permian in Xiangzhong depression as an example. *Earth Science Frontiers* **2016**, 23, (01), 44-53.

28. Zhang, J.Z.; Li, X.Q.; Liu, Y.; Cai, Y.Q.; Wang, Y.; Niu, H.Y.; Hu, S.L.; Xu, X., Longtan Formation Shale Gas Reservoiring Conditions and Favorable Region Analysis in Southern Sichuan Area. *Coal Geology of China* **2014**, 26, (12), 1-6.

29. Guo, X.S.; Hu, D.F.; Liu, R.B.; Wei, X.F.; Wei, F.B., Geological conditions and exploration potential of Permian marine–continent transitional facies shale gas in the Sichuan Basin. *Natural Gas Industry* **2018**, 38, (10), 11-18.

30. Chen, F.R.; Wei, X.F.; Liu, Z.J.; Ao, M.C.; Yan, J.H., Pore development characteristics and main controlling factors of the Permian marine-continent transitional shale in the Sichuan Basin. *Natural*

Gas Geoscience **2020**, 31, (11), 1593-1602.

31. Cao, Q.; Jiang, K.; Wen, Z.; Wang, X.; Qi, M.; Yin, Z.; Brogi, A., Characteristics of Organic Matter Pores and the Relationship with Current Pressure System of Lower Silurian Longmaxi Shales in Dingshan Field, Southern Sichuan, China. *Geofluids* **2021**, 2021, 9967479.
32. Cao, Q.; Wen, Z.T.; Xu, H.; Qi, M.H.; Wang, X.Z., Development characteristics and influencing factors of organic matter pores in Longmaxi Formation shale in D area, southern Sichuan Basin, China. *Journal of Chengdu University of Technology (Science & Technology Edition)* **2021**, 48, (05), 599-609+625.
33. Jiang, K.; Zhou, W.; Deng, N.; Song, W., Statistical analysis and significance of pyrite in the Wufeng-Lower Longmaxi Shale Formation in South China. *Arab J Geosci* **2020**, 13, (22), 1-18.
34. Wang, W.; Shi, W.B.; Fu, X.P.; Chen, C., Shale gas exploration potential and target of Permian Dalong Formation in northern Sichuan. *Petroleum Geology ＆ Experiment* **2020**, 42, (06), 892-899+956.
35. Yang, X.; Fang, K.; Luo, P.; Zhao, S.M.; Ye, D.N., Geological characteristics and gas bearing capacity of shale from transitional marine-continental facies of Longtan Formation: A case study from the Well LD1 in Gulin-Xuyong, southern Sichuan. *Sedimentary Geology and Tethyan Geology* **2021**, 41, (03), 446-453.
36. Nesbitt, H.W.; Young, G.M., Early Proterozoic climates and plate motions inferred from major element chemistry of lutites. *Nature* **1982**, 299, (5885), 715-717.
37. Huang, Z.S.; Wang, X.Z.; Yang, X.Y.; Zhu, R.K.; Cui, J.W.; Lu, Y.Z.; Li, Y., Constraints of Sedimentary Environment on Organic Matter Accumulation in Shale: A case study of the Wufeng-Longmaxi Formations in the southern Sichuan Basin. *Acta Sedimentologica Sinica* **2021**, 39, (03),

631-644.

38. Cao, Q.; Zhou, W.; Deng, H.; Chen, W., Classification and controlling factors of organic pores in continental shale gas reservoirs based on laboratory experimental results. *J Nat Gas Sci Eng* **2015**, 27, 1381-1388.

39. Ma, R.Y.; Zhang, J.; Wang, M.; Ma, W.P.; Zhao, J.G., Micro-pore characteristics and gas-bearing properties of marine continental transitional shale reservoirs in the Qinshui basin. *Journal of Henan Polytechnic University (Natural Science)* **2021**, 40, (04), 66-77.

40. Song, K.L.; Yao, L., Study on the characteristics of gas-generating reservoir-caprock assemblages and gas traps in coal measures in Fuxin Basin. *IOP conference series. Earth and environmental science* **2020**, 558, (2), 22002.

41. Ma, X.; Guo, S.B., Comparative Study on Shale Characteristics of Different Sedimentary Microfacies of Late Permian Longtan Formation in Southwestern Guizhou, China. *Minerals-Basel* **2019**, 9, (1), 20.

42. Min W.; Yang J.; Wang Z.; Lu S., Nanometer-Scale Pore Characteristics of Lacustrine Shale, Songliao Basin, NE China[J]. Plos One, 2015, 10.

43. Beauchamp, B.; Baud, A., Growth and demise of Permian biogenic chert along northwest Pangea: evidence for end-Permian collapse of thermohaline circulation. *Palaeogeography, Palaeoclimatology, Palaeoecology* **2002**, 184, (1-2), 37-63.

Figures and Tables
Click here to access/download;Figure;table+figure-2021.12.pdf

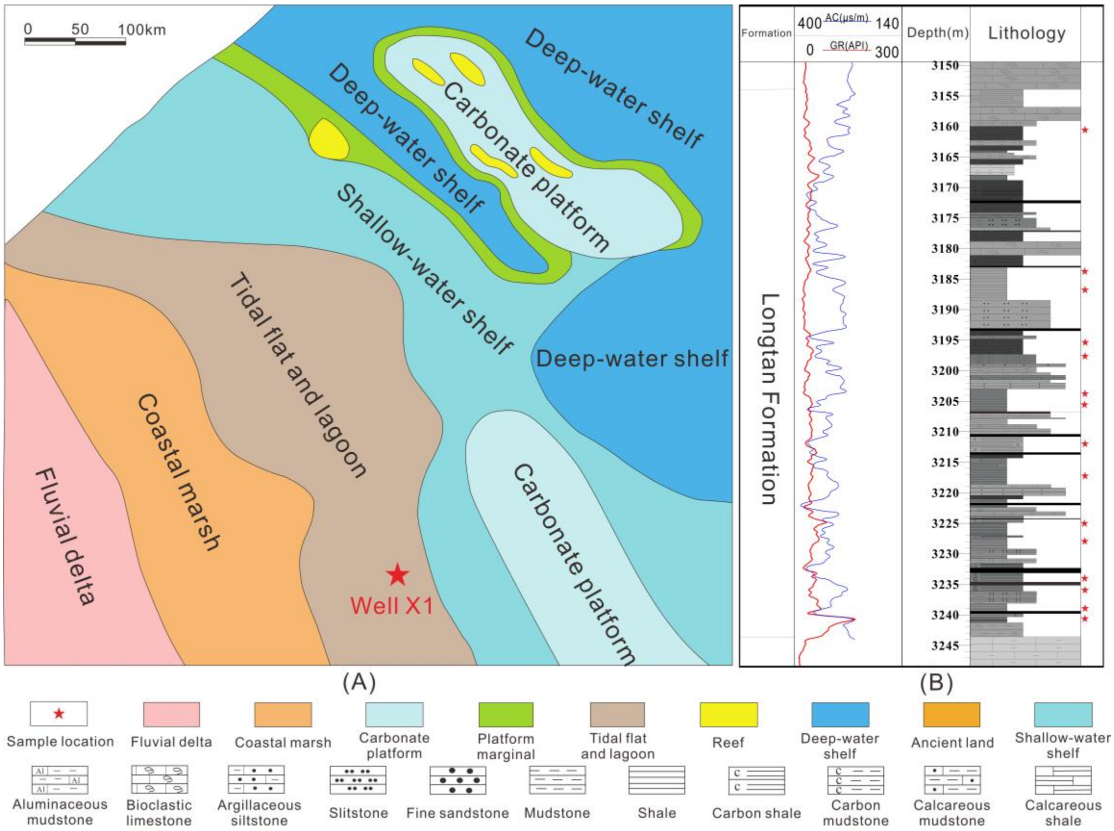

Figure 1. (a) Sample location; (b) Well X1 in Longtan Formation (modified from [29] and [30])

Table 1. Comparison of lithological combinations in Permian Longtan Formation, Well X1, southeastern Sichuan Basin

Formation	Upper Longtan	Middle Longtan	Lower Longtan
Depositional model	Delta	Tidal flat and lagoon, developing swamp facies in the top	Tidal flat and lagoon, developing swamp facies in the bottom
Subfacies	Delta	Lagoon facies mainly, part tidal flat-swamp facies	Developing tidal flat- swamp facies, part of the lagoon facies
Microfacies	Peat swamp	Lagoon mud and intertidal mixed flat, peat swamp	Tidal flat-peat swamp, lagoon mud
Lithological combination	Limestone with bio-clastic, Siltstone with mud, Silty shale, Occasional thin coal seam	Thick shale with limestone and silty shale mainly, part of shale and sandstone interbedded, and developing coal seam and nodular siderite	Shale interbedded with siltstone and limestone mainly, developing thick carbonaceous shale and thick coal seam, and siderite distributed as granular and clumpy
Microscopic characteristics	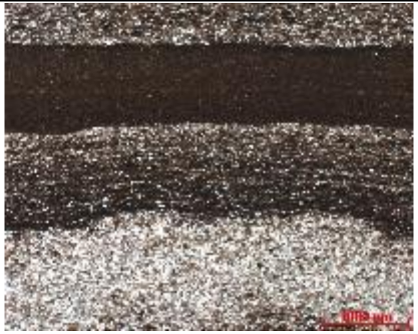	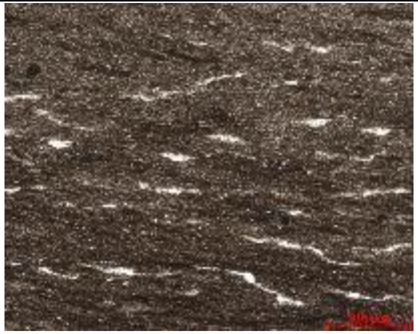	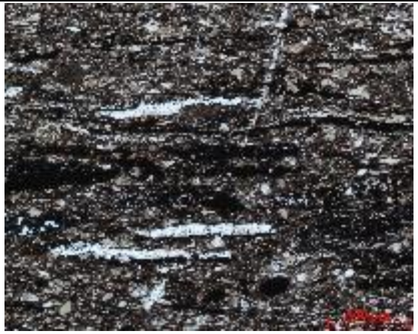
	Sample 3#: Argillaceous siltstone. The aggregation of clay minerals is mainly banded, and organic matter is clumpy and fine banded, 5×10 (-).	Sample 7#: Shale. Organic matter are distributed along the bedding, 5×10 (-).	Sample 9#: Silty shale. Silty lamination develops, and organic matter is black speckled, clumpy and banded, 5×10 (-).

	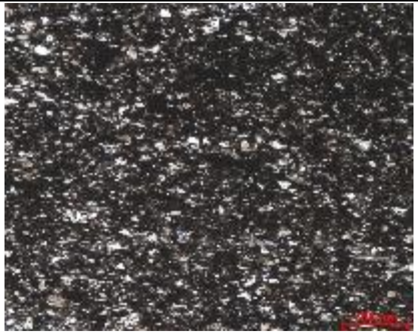	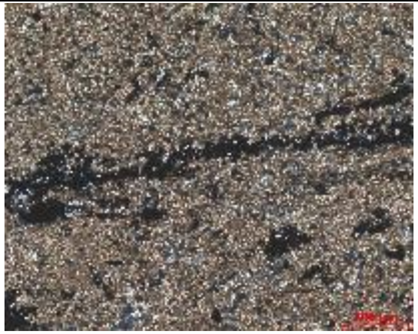	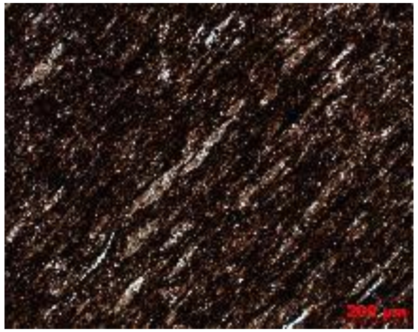
	Sample 3#: Siltstone with mud. Organic matter is black clumpy and banded, 5×10 (-).	Sample 4#: Shale with dolomite. Organic matter develops bedding, 10×10 (+).	Sample 6: Shale with siltstone. Bedding development, and organic matter develops bedding, 10×10 (-).
	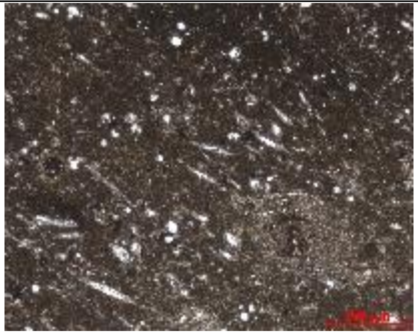	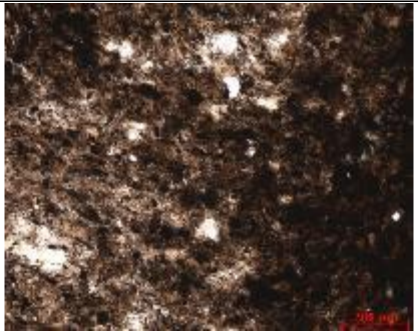	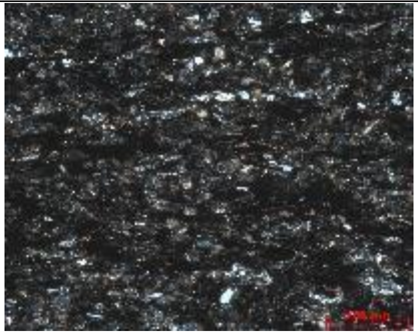
	Sample 3#: Shale with bioclasts. Bioclasts are mainly spongy spicules scattered in rocks in needle-like and elliptical shapes, 5×10 (+).	Sample 8#: Carbonaceous mudstone. The mudstone is yellow-brown in color and is mainly an aggregation of microcrystalline and cryptocrystalline clay minerals. A few cracks are filled with organic matter, 10×10 (-).	Sample 9#: Silty shale, 5×10 (+).

	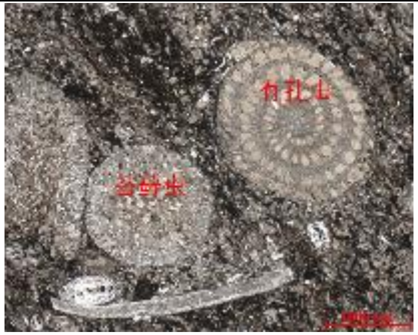	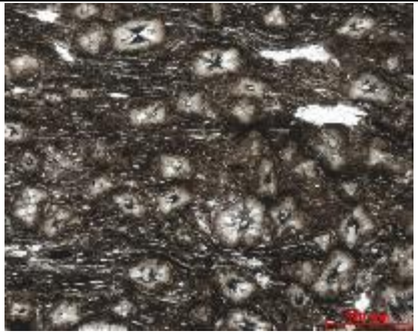	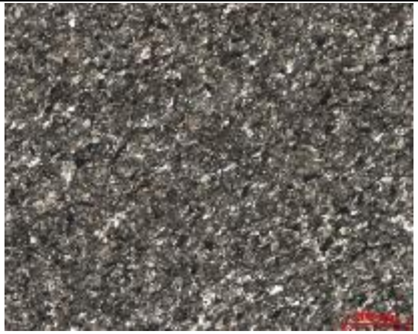
	Sample 14#: Bioclastic limestone. Bioclasts such as foraminifers and bryozoans were distributed elliptically, most of which were metasomatized by calcite and siliceous. A small number of ostracoda fragments were found scattered in the shape of tiny crescent, 5×10 (+).	Sample 6#: Mudstone. Siderite is nodular, spherical, and has a radial structure inside, with the overall characteristics of orthogonal extinction with sizes ranging from 0.1–1.0 mm, 10×10 (+).	Sample 9#: Argillaceous limestone. Siderite mainly consists of granular aggregates with fine microcrystals, and the whole aggregates are granular and clumpy, 5×10 (+).
Sedimentary structure	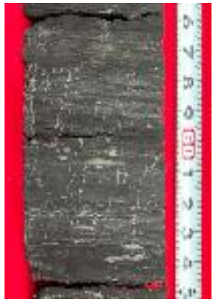	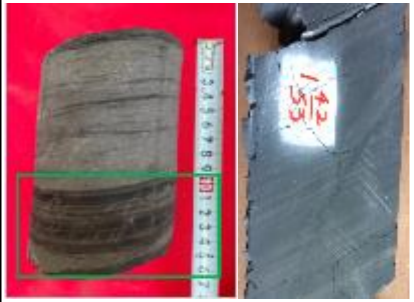	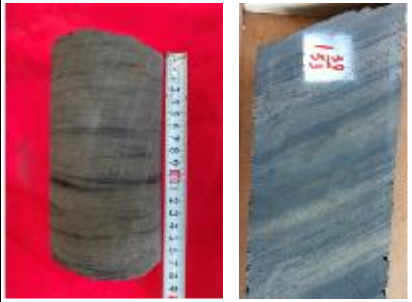
Bedding type	Developing bivalves and bryophytes Bioturbation, horizontal bedding, nodular siderite.	Horizontal bedding, wave-ripple bedding.	Lenticular bedding, veined bedding, cross bedding.

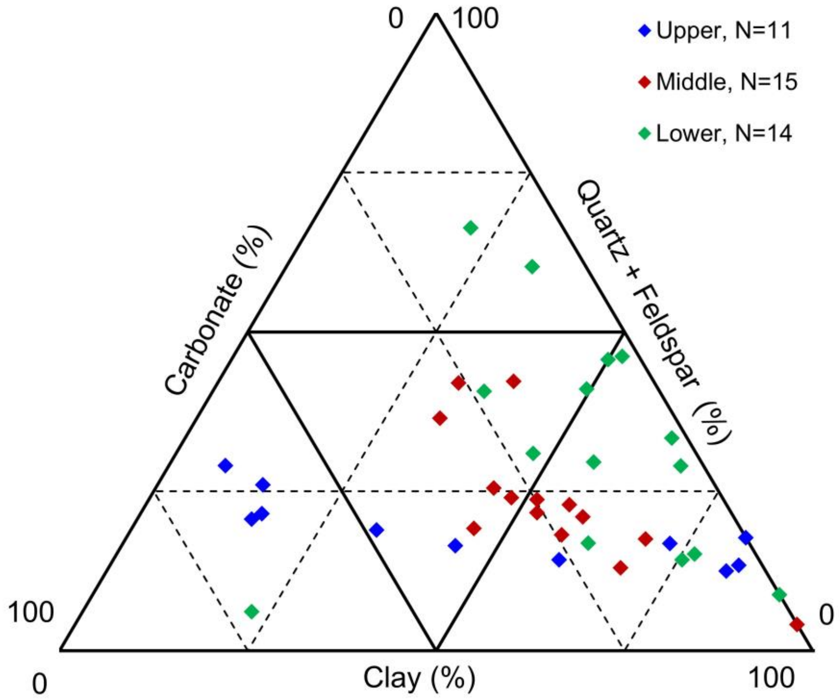

Figure 2. Triangulation of mineral composition of Permian Longtan Formation samples from southeastern Sichuan (Note: the datum is from this research and Sinopec Southwest Exploration and Development Research Institute)

Table 2.Analysis results of kerogen type of Longtan Formation samples (transmitted light + fluorescent)

Sample	Formation	Lithology	Sapropel (%)	Exinite (%)	Vitrinite (%)	Inertinite (%)	Type Index	Type
2#	Longtan Formation	Calcareous mudstone	-	66	28	6	6.00	II_2

3#	Longtan Formation	Carbonaceous mudstone	-	62	33	5	1.25	II_2
4#	Longtan Formation	Mudstone with limestone	-	42	53	5	-23.75	III
6#	Longtan Formation	Mudstone	-	54	41	5	-8.75	III
7#	Longtan Formation	Silty mudstone	-	54	41	5	-8.75	III
8#	Longtan Formation	Mudstone	-	23	71	6	-47.75	III
10#	Longtan Formation	Silty mudstone	-	64	32	4	4.00	II_2
11#	Longtan Formation	Carbonaceous mudstone	-	30	65	5	-38.75	III
13#	Longtan Formation	Argillaceous siltstone	-	66	30	4	6.50	II_2
14#	Longtan Formation	Shale	-	74	24	2	17.00	II_2
15#	Longtan Formation	Mudstone with siltstone	-	82	16	2	27.00	II_2

Note: “-” indicates that the contents are negligible.

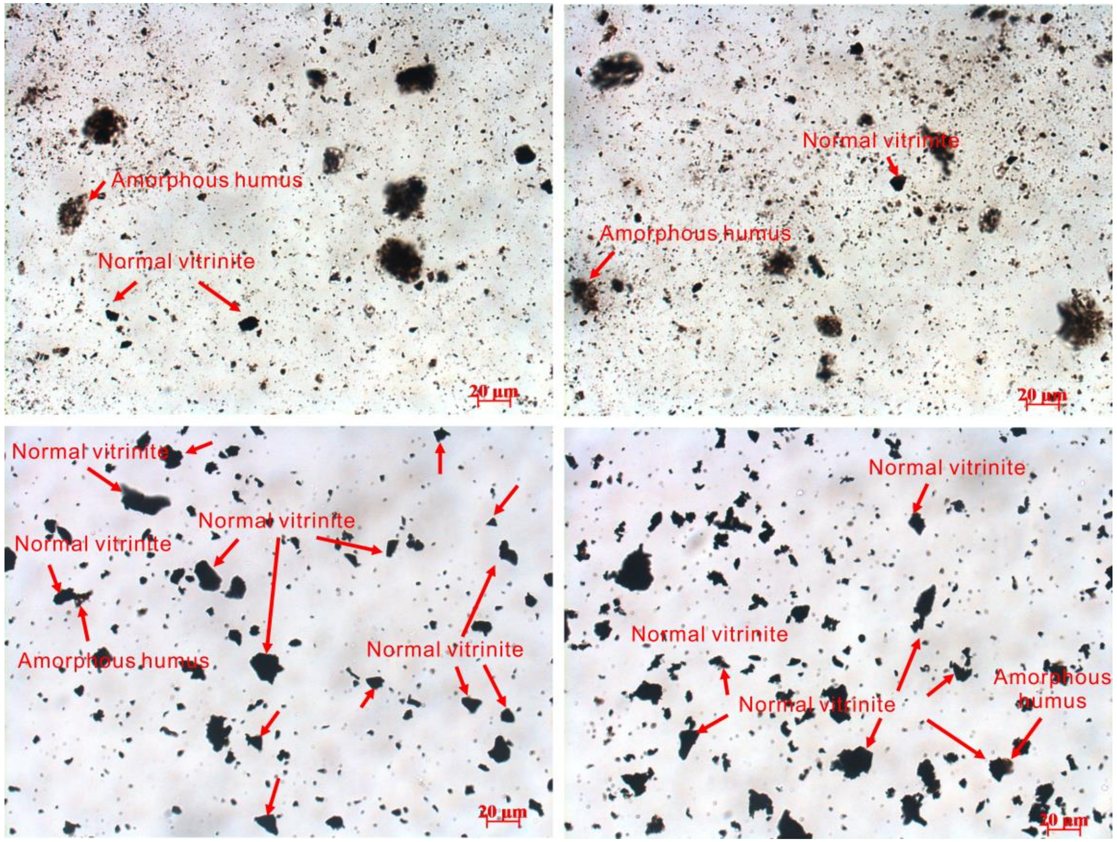

Figure 3. Organic photomicrographs of typical shale sample in Longtan Formation under fluorescent

Table 3.Major element oxide contents of different samples of Longtan Formation in study area

Sample number \ Minimum detectable value	Major element oxide contents (%)										Ignition loss (%)	CIA(g/mol)
	SiO_2	Al_2O_3	MgO	Na_2O	K_2O	P_2O_5	TiO_2	CaO	TFe_2O_3	MnO		
	0.0335	0.0375	0.0277	0.0021	0.0382	0.0019	0.0333	0.0438	0.0078	0.0138	/	83.97
1#	40.51	13.47	2.26	0.52	1.84	0.17	2.60	11.52	8.02	0.10	18.34	84.01
2#	33.57	14.16	2.89	0.74	0.91	0.31	2.83	7.73	15.89	0.15	18.01	79.60
3#	42.62	24.25	0.83	1.07	1.34	0.33	4.57	0.70	6.60	0.02	17.37	84.86
4#	38.04	23.34	1.02	1.10	0.90	0.21	5.08	0.75	12.43	0.17	16.62	83.78
5#	43.35	24.07	1.33	1.10	0.96	0.36	4.57	1.58	7.53	0.06	14.22	80.87
6#	40.30	23.19	1.15	1.45	1.40	0.40	4.79	0.86	9.58	0.11	16.32	81.94
7#	41.71	15.07	3.43	0.63	0.48	0.37	4.20	5.46	16.22	0.09	9.81	81.20
8#	35.27	10.01	3.83	0.15	0.20	0.43	1.70	11.80	19.84	0.16	15.00	81.72
9#	40.64	19.15	2.12	1.38	1.32	0.42	3.11	4.12	11.90	0.16	14.80	83.56
10#	36.34	12.73	2.61	0.59	0.52	0.18	1.59	1.08	28.45	0.30	15.81	77.85
11#	40.92	19.39	1.74	1.14	1.63	0.41	3.66	2.48	12.28	0.16	15.44	79.90
12#	38.92	23.07	1.09	0.87	1.97	0.06	4.10	0.62	14.45	0.04	14.47	82.84
13#	39.58	22.98	0.66	1.17	1.46	0.21	3.78	0.69	10.15	0.20	19.53	79.69
14#	41.36	25.39	0.47	0.62	0.83	0.23	4.55	0.50	6.06	0.11	20.11	79.00
15#	37.94	19.62	1.35	0.59	3.02	0.45	3.36	3.51	14.37	0.15	13.11	81.10

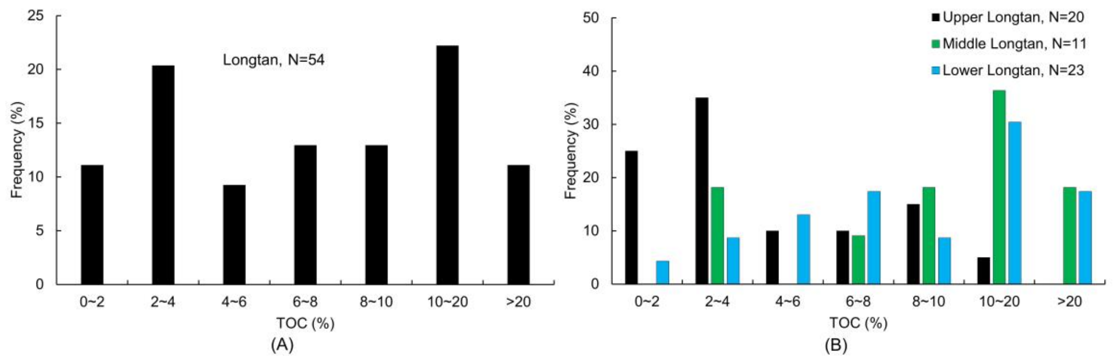

Figure 4. The distribution of TOC contents of Longtan Formation samples (the datum are from this research and reference [13, 15, 30])

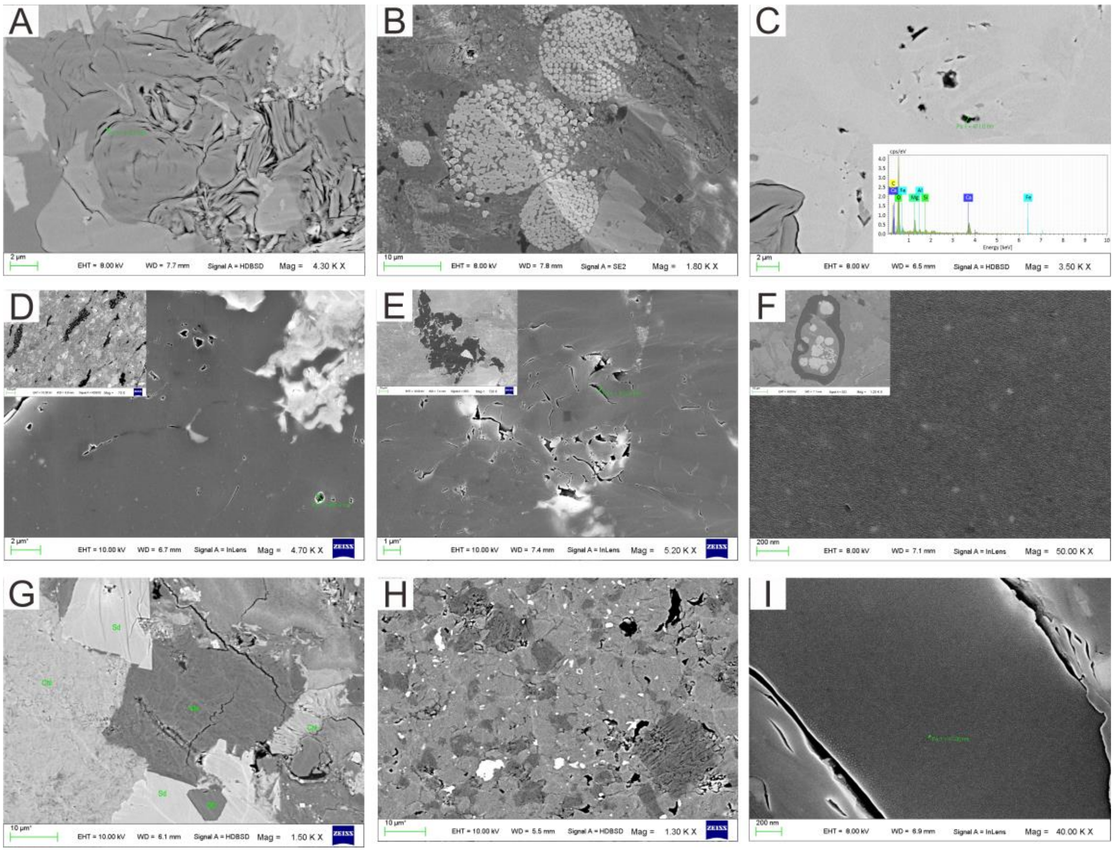

Figure 5. Distribution characteristics of microscopic reservoir space in different shale samples of the Longtan Formation

A. Sample 8#: interlayer fractures developing in lamellar clay minerals (×4300); B. Sample 2#: Framboidal pyrite aggregations with parts of the intercrystal pore are filled with clay minerals (×1800); C. Sample 5#: Irregular dissolution pores in siderite (confirmed by energy spectrum)

(×3500); D. Sample 9#: The sample structure is tight, clastic interbedded with clay minerals and organic matter, and the organic matter is fragmentary or interstitial (×70). Irregular pores and microfractures are developed in fragmentary organic matter (×4700); E. Sample 12#: Interstitial organic matter interbedded with clay minerals and siderite, and developing pores and fractures (×5200); F. Sample 4#: The interior of crust organic matter is filled with micro-granular and framboidal pyrite, and clay minerals (×1200) with little pores (×50000); G. Sample 9: Microfractures (×1500); H. Sample 12#: Micro-granular quartz in inlaying contact interbedded with lamellar kaolinite aggregations; intergranular pores and fractures develop (×1300); I. Sample 14: The pores in banded organic matter are not developed, but shrinkage fractures develop at the edge of organic matters (×40000).

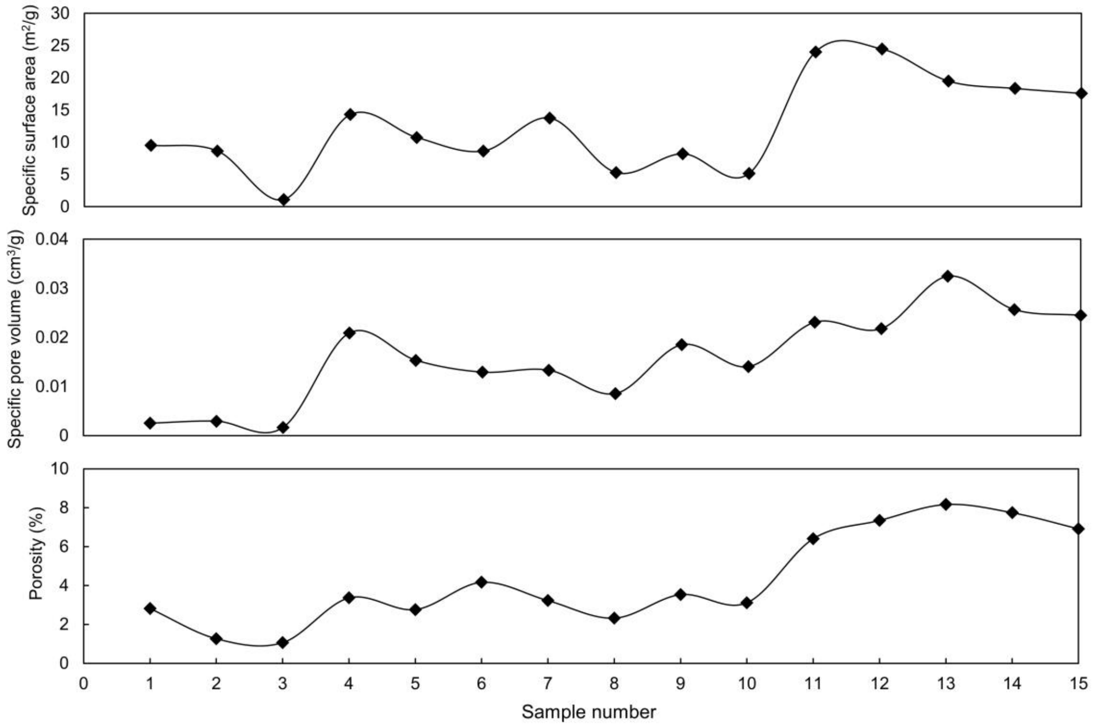

Figure 6. The distributions of porosity, specific surface area and specific pore volume of different samples

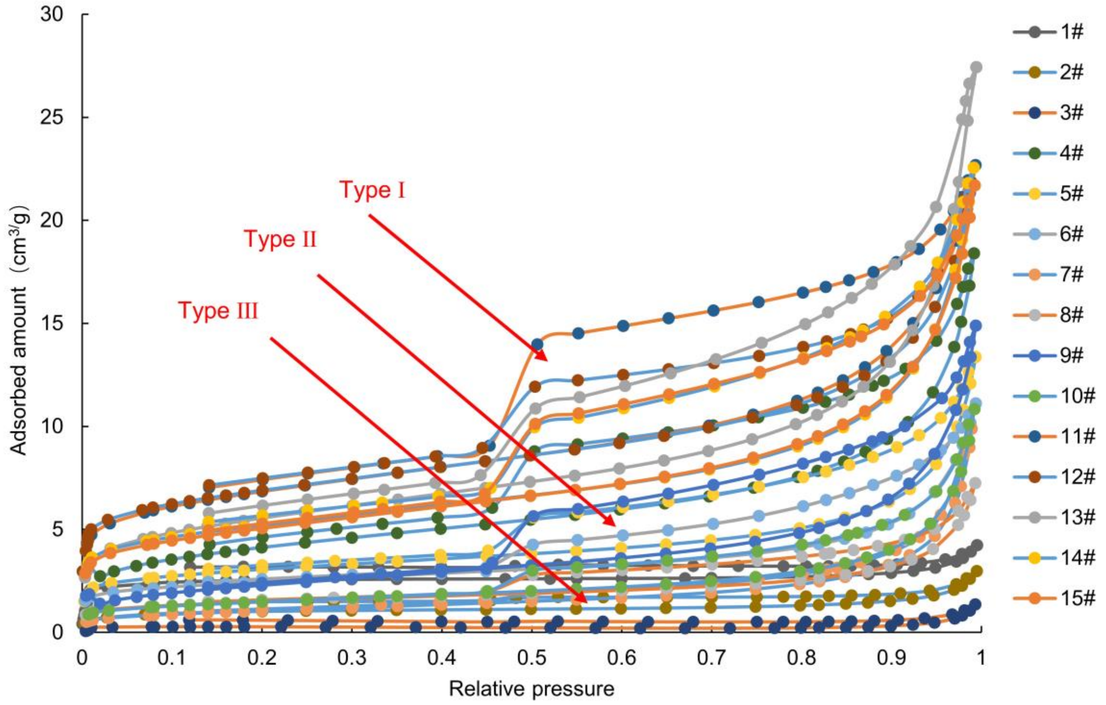

Figure 7. Isotherm adsorption and desorption curves of low temperature nitrogen of different samples

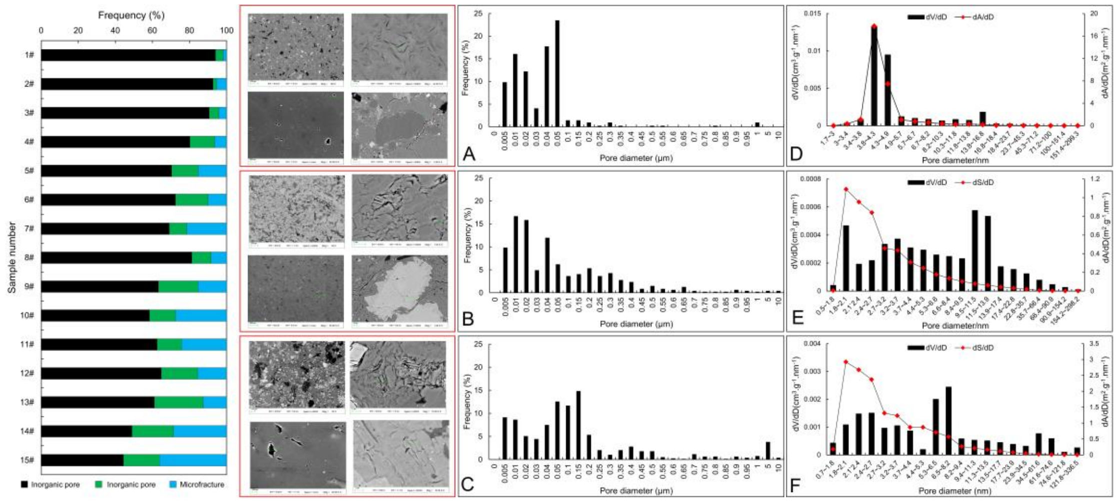

Figure 8. Pore types and structure characteristic parameters in different shale by SEM and image analysis

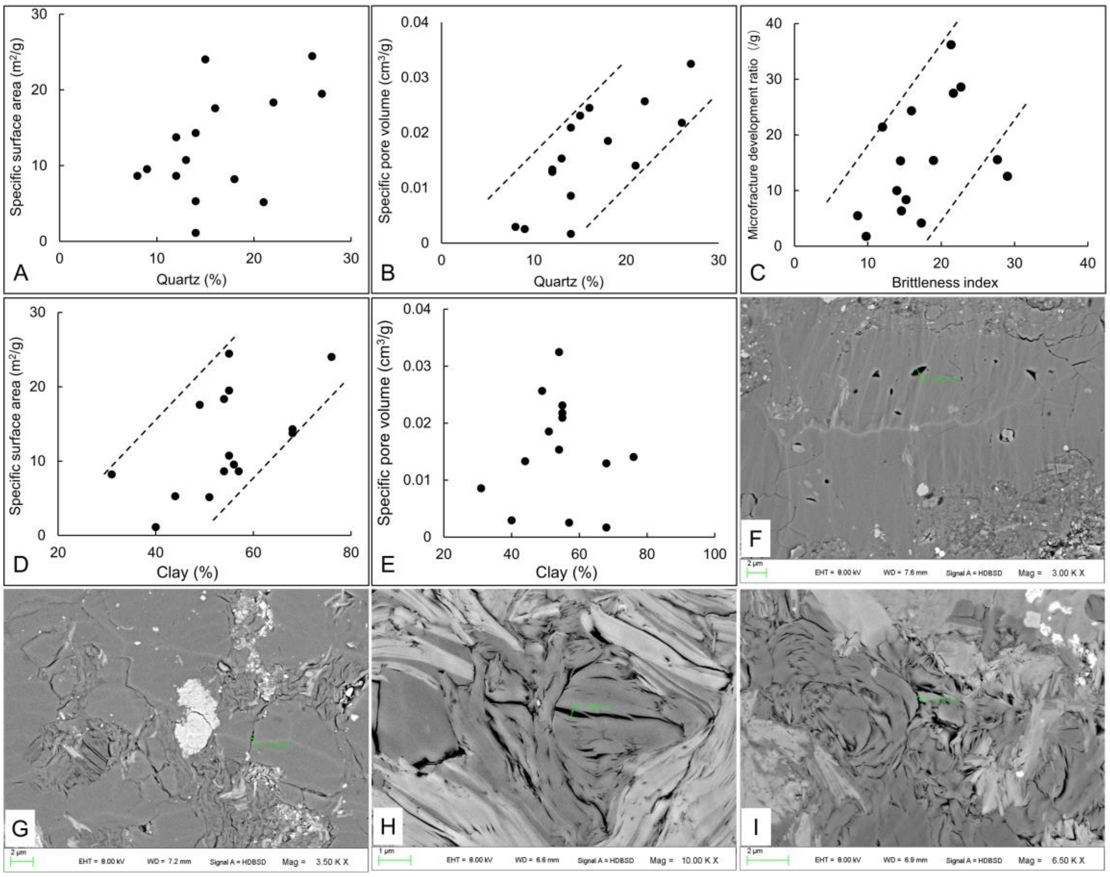

Figure 9. The relationship between the different mineral components (quartz, clay minerals) and pore volume, specific surface area and microfracture development ratio

A. The relationship between the quartz and specific surface area; B. The relationship between the quartz and specific pore volume; C. Correlation between microfracture development ratio and brittleness index; D. The relationship between the clay minerals and specific surface area; E. The relationship between the clay minerals and specific pore volume; F. An irregular pore and microcrack in microgranular quartz (×3000); G. Particle margin fractures in microcrystalline quartz (siliceous) (×3500); H. Interbedded fracture in lamellar andreattite aggregation (×10000); I. Interlayer pores in alternately distributed lamellar andreattite and chlorite aggregation (×6500)

Click here to access/download
Supporting Information
Funding.docx
