# Supplementary material for: Effect of different lithological assemblages on shale reservoir properties in the Permian Longtan Formation, southeastern Sichuan Basin: Case study of Well X1
Source: PLoS One. 2022 Aug 12;17(8):e0271024. doi: 10.1371/journal.pone.0271024 (PMC9374248; doi:10.1371/journal.pone.0271024)
Supplement: Attachment — Submitted filename: Response to Editors and Reviewers.docx [file pone.0271024.s008.docx]

Dear Editors and Reviewers,

On behalf of my co-authors, we thank you very much for giving us an opportunity to revise our manuscript, we appreciate editor and reviewers very much for their positive and constructive comments and suggestions on our manuscript entitled “properties in the Permian Longtan Formation, southeastern Sichuan Basin: Case study of Well X1”. (ID: PONE-D-21-40287).

We have studied reviewers’ comments carefully and have made revisions that are marked in red in the paper. We have tried our best to revise our manuscript according to the comments. Attached please find the revised version, which we would like to submit for your kind consideration.

We would like to express our great appreciation to you and the reviewers’ comments on our paper. Looking forward to hearing from you.

Thank you and best regards.

Yours sincerely,

Dr. Qian Cao

State Key Laboratory of Shale Oil and Gas Enrichment Mechanisms and Effective Development

Response to editors，

1. Thank you for updating your data availability statement. You note that your data are available within the Supporting Information files, but no such files have been included with your submission. At this time we ask that you please upload your minimal data set as a Supporting Information file, or to a public repository such as Figshare or Dryad.
   Please also ensure that when you upload your file you include separate captions for your supplementary files at the end of your manuscript.
   As soon as you confirm the location of the data underlying your findings, we will be able to proceed with the review of your submission.

Reply: Fig 1 in our manuscript is from CHEN F.R. et al.[30], and we have not modified the content of this figure (Fig 1 “Sedimentary facies distribution of Longtan Formation/Wujiaping Formation in Sichuan Basin and its periphery” ). We were added the sampling location and stratigraphic description. At present, we have contacted the editor of Natural Gas Geoscience and obtained the Copyright permission that allows us to cite this figure in the article. The Copyright Permission is listed as follows.

All relevant data are within the paper. We have uploaded our data set as a Supporting Information file“data of Fig3,5,7,8,910. excel”, and we also have listed the separate captions for our supplementary files at the end of our manuscript. If this data display format does not meet the requirements of journals, please inform me.

2. We note that the grant information you provided in the ‘Funding Information’ and ‘Financial Disclosure’ sections do not match.

Reply: The author(s) received no specific funding for this work.

3. In your Methods section, please provide additional information regarding the permits you obtained to collect samples for the present study. Please ensure you have included the full name of the authority that approved the field site access and, if no permits were required, a brief statement explaining why.

Reply: This work was carried out in State Key Laboratory of Shale Oil and Gas Enrichment Mechanisms and Effective Development, and the sampling and experiments of "X1 well" are allowed in the research process. And the samples were from the State Key Laboratory of Shale Oil and Gas Enrichment Mechanisms and Effective Development, which is the first institution of the present study. There is no need to provide additional information regarding the permits we obtained to collect samples for the present study.

Response to Reviewers

1. I realize that the authors have a misleading in the meaning of Formation, i.e., in the Abstract section, the authors state that “the lithological development divides the Longtan Formation into lower (swamp), middle (tidal flat/lagoon) and upper (delta) sub-members” whereas, on the manuscript (section 3.1), the authors state that “the differences in the lithological assemblages in showed that Longtan Formation could be further divided into the Lower, Middle and Upper Longtan Formations.” From a stratigraphy point of view, a formation can be divided into members and grouped together in groups. Please, modify to lower, middle, and upper members.

Reply: By relearning the meaning of “Formation” that a formation can be divided into members and grouped together in groups, the description of Longtan Formation has been reorganized from a stratigraphy point of view in the paper. The Longtan Formation was divided into the lower, middle, and upper members due to the different lithological development. This part of the description has also been modified throughout the article. And all the amendments are highlighted in red in the revised manuscript.

2. The main issue is the writing style that is quite poor in places and needs careful and thorough review to improve the clarity, conciseness, grammar, sentence structure, and organization. I accept that the authors may not be native English speakers, but the text would benefit from additional editing. I suggest the authors significantly polish the English or get the help of a native speaker. Some sentences are too long and entire paragraphs are too confusing for the reader. It should be made both more concise and sharper.

Reply: The language of our manuscript have been refined and polished by International Science Editing. The supporting documents are as follows:

3. The introduction should provide a better geographical context for readers not familiar with the study area.

Reply: We have described the geographical context of the study area in detail in Chapter 2.1 of the article, including the changes of sedimentary environment, stratigraphic distribution, etc. line 40-46, line 51-64, and line 82-93.

4. I encourage the authors to consider citing the figures of the manuscript (i.e., figure 7 is not cited at all on the manuscript).

Reply: We have checked the descriptions of all graphs and tables in the article in detail, and each graph and table is reasonably referenced.

5. Figure captions are inadequate. Figure captions should be comprehensive but concise; they should clearly describe the contents of the figure; they should draw attention to key features in the figure. Figure captions should provide enough information so that the reader can easily review the figure without referring to the text. So, I would ask the authors to rewrite the figure captions.

Reply: We have tried our best to rewrite the figure captions according to the comments, including Figures 1, 2, 5, 6, 10 in Supporting information, and line 512 - 583.

6. In figure 1, the authors should add a general map to better show the southeaster Block of the Sichuan Basin in Southern China and cite it in the introduction (section 1). In figure 1b, I motivate the authors to add the samples label of the 15 collected samples to show the exact stratigraphic position of each sample in the well-log. Always in figure 1b, it is too difficult to see the lithologies in the well-log.

Reply: We have added a general map to identify the location of Sichuan Basin, this figure is explained in the introduction (line 40-46, and line 53-55). Besides, the lithology and other information of the sample are also recorded when collecting these samples, and the lithologies labels of these 15 samples have been identified in Figure 1B. It is hoped that this description will enable readers to clearly see the information of the collected samples.

We tried our best to improve the manuscript and made some changes in the manuscript. These changes will not influence the content and framework of the paper. And here we have marked the changes in red in revised paper.

We appreciate for editors and reviewers’ warm work earnestly, and hope that the correction will meet with approval.

Once again, thank you very much for your comments and suggestions.
